# 4′-Deoxypyridoxine disrupts vitamin B_6_ homeostasis in *Escherichia coli* K12 through combined inhibition of cumulative B_6_ uptake and PLP-dependent enzyme activity

**DOI:** 10.1099/mic.0.001319

**Published:** 2023-04-11

**Authors:** Jill Babor, Angela Tramonti, Caterina Nardella, Adam Deutschbauer, Roberto Contestabile, Valérie de Crécy-Lagard

**Affiliations:** ^1^​ Department of Microbiology and Cell Science, University of Florida, Gainesville, FL 32611, USA; ^2^​ Istituto di Biologia e Patologia Molecolari, Consiglio Nazionale delle Ricerche, Roma, Italy; ^3^​ Istituto Pasteur Italia - Fondazione Cenci Bolognetti, Dipartimento di Scienze Biochimiche, Sapienza Università di Roma, Rome, Italy; ^4^​ Environmental Genomics and Systems Biology Division, Lawrence Berkeley National Laboratory, Berkeley, CA 74720, USA; ^5^​ University of Florida Genetics Institute, Gainesville, FL 32610, USA

**Keywords:** Pyridoxal 5′-phosphate, anti-metabolite, competitive inhibition

## Abstract

Pyridoxal 5′-phosphate (PLP) is the active form of vitamin B_6_ and a cofactor for many essential metabolic processes such as amino acid biosynthesis and one carbon metabolism. 4’-deoxypyridoxine (4dPN) is a long known B_6_ antimetabolite but its mechanism of action was not totally clear. By exploring different conditions in which PLP metabolism is affected in the model organism *Escherichia coli* K12, we showed that 4dPN cannot be used as a source of vitamin B_6_ as previously claimed and that it is toxic in several conditions where vitamin B_6_ homeostasis is affected, such as in a B_6_ auxotroph or in a mutant lacking the recently discovered PLP homeostasis gene, *yggS*. In addition, we found that 4dPN sensitivity is likely the result of multiple modes of toxicity, including inhibition of PLP-dependent enzyme activity by 4’-deoxypyridoxine phosphate (4dPNP) and inhibition of cumulative pyridoxine (PN) uptake. These toxicities are largely dependent on the phosphorylation of 4dPN by pyridoxal kinase (PdxK).

## Introduction

Pyridoxal 5′-phosphate (PLP) is one of six interconvertible B_6_ vitamers and is the most common catalytically active form of vitamin B_6_ (B_6_). PLP plays an essential role as a cofactor for a wide variety of metabolic enzymes [1]. All known organisms, even those with minimal genomes, harbour PLP-dependent enzymes, and therefore require PLP for survival [2]. While essential, PLP is also highly reactive and labile, making free cellular PLP susceptible to damage and degradation, as well as toxic at high concentrations [3]. This dilemma raises important questions about how the essential vitamer can be made available to the numerous enzymes requiring it as a cofactor, while still being maintained at low enough concentrations to prevent cellular damage and toxicity [4].

**Fig. 1.**
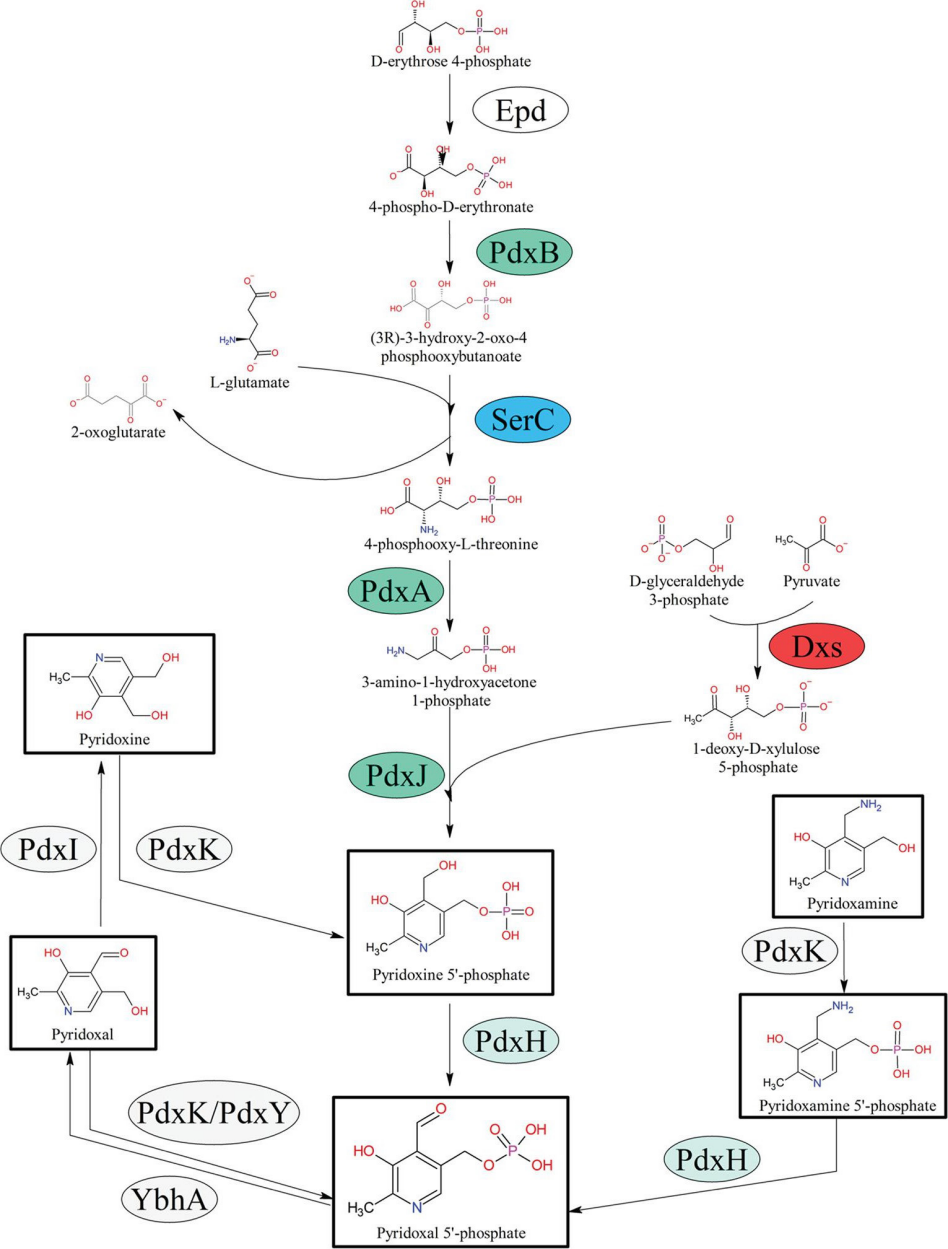
*
E. coli* pyridoxal 5′-phosphate biosynthesis and salvage. The *E. coli* DXP-dependent *de novo* PLP biosynthesis and salvage pathways are displayed by solid black arrows with different forms of vitamin B_6_ boxed in black. All enzymes are presented in ovals. Enzymes whose loss of activity result in vitamin B_6_ auxotrophy are coloured seafoam green, with auxotrophs of multiple pathways coloured in blue. Dxs (red) is an essential enzyme in *E. coli* and cannot be deleted. Enzymes of the PLP salvage pathway have chequered backgrounds.

Wild-type *Escherichia coli* is able to synthesize PLP *de novo* utilizing the deoxyxylulose 5′-phosphate (DXP)-dependent PLP biosynthesis pathway [5] (Fig. 1). Several mutants of the *de novo* PLP biosynthesis pathway are auxotrophic for vitamin B_6_: Δ*pdxA*, Δ*pdxB*, Δ*pdxH*, Δ*pdxJ*, and Δ*serC* [6]. These auxotrophs are able to survive by utilizing the PLP salvage pathway to take up B_6_ vitamers from the environment and convert them to PLP through the activity of PdxH and PdxK salvage enzymes, pyridoxine 5′-phosphate oxidase and pyridoxine/pyridoxal/pyridoxamine kinase, respectively [5]. While PdxK can act on all non-phosphorylated B_6_ vitamers [pyridoxal (PL), pyridoxine (PN) and pyridoxamine (PM)] a second pyridoxal-specific kinase, PdxY, exists in *E. coli* and converts PL to PLP directly [7] (Fig. 1). Two additional salvage enzymes, PdxI and YbhA, are also important for interconversion between B_6_ vitamer species and ultimately maintaining appropriate levels of PLP. PdxI has been annotated as a pyridoxine 4-dehydrogenase and acts mainly to convert PL to PN (as a pyridoxal reductase) when taken up from the environment or media [8, 9]. YbhA has been annotated as a pyridoxal 5′-phosphate phosphatase and carries out the dephosphorylation of PLP to PL [10] (Fig. 1).

Genes encoding B_6_ transporters have yet to be identified in most organisms, including *E. coli *, but pioneering literaure in the field and the ability of auxotrophs to salvage B_6_ vitamers from the environment suggest that they must exist. *E. coli* transports only the non-phosphorylated vitamers in a rapid, energy-dependent process that likely involves at least two transporters that vary by substrate specificity. While uptake of the vitamers is likely through transporter-dependent facilitated diffusion, it appears that cumulative uptake of B_6_ is driven by subsequent phosphorylation [11–13].

Though many PLP-dependent enzymes have been well studied due to the strong link between PLP imbalance and human disease states, the mechanism behind maintaining an appropriate balance of B_6_ vitamers is not well understood in any organism [5, 14, 15]. One such PLP-dependent protein, YggS of *E. coli *, belongs to the highly conserved COG0325 family of proteins. COG0325 proteins have recently been implicated as playing a major role in maintenance of vitamin B_6_ (PLP) homeostasis from *E. coli* to humans [4, 16], leading to naming members of this family ‘Pyridoxal phosphate homeostasis proteins’ or PLPHPs. To date, no enzymatic function has been identified for YggS or its homologs, but an *E. coli yggS* knockout mutant has significantly altered intracellular amino acid pools [17] and exhibits a PN sensitivity phenotype that likely results from the phosphorylation of imported PN and subsequent accumulation of intracellular pyridoxine 5′-phosphate (PNP) [18].

4-Deoxypyridoxine (4dPN) is a PN analogue and vitamin B_6_-antimetabolite that has been reported to competitively inhibit uptake of extracellular B_6_ [12, 19]. Additionally, pioneering studies from the 1950–80s showed that 4dPN was transported into many cell types, including *E. coli* cells, and that its presence inhibited the activity of PLP-dependent enzymes [19]. Hence, 4dPN seems an ideal compound to perturb PLP-related processes and to probe specific physiological functions of PLP-dependent enzymes and their respective metabolic pathways. By analysing the response of wild-type *E. coli* K12 and mutants of the PLP synthesis, salvage and homeostasis pathways to 4dPN exposure, we confirm in the current study that this antimetabolite inhibits cumulative B_6_ salvage through competitive inhibition of PdxK kinase activity after uptake of exogenous vitamers. We also found that the *yggS* mutant was extremely sensitive to 4dPN through the formation of 4dPNP, and that this observed toxicity was caused by a synergistic negative inhibition of PLP enzymes by both PNP and 4dPNP. The additional findings reported in this study highlight the complexity of physiological vitamin B_6_ homeostasis and can be used to guide future studies in the field.

## Methods

### Growth conditions and media

Bacterial cultures were grown in LB medium (tryptone 10 g l^− 1^, yeast extract 5 g l^− 1^, NaCl 10 g l^− 1^) or M9 0.4 % glucose minimal medium [20] at 37 °C, unless otherwise specified. Growth media were solidified with agar (16 g l^− 1^) for the preparation of plated media. LB Lennox medium (tryptone 10 g l^− 1^, yeast extract 5 g l^− 1^, NaCl 5 g l^− 1^, agar 16 g l^− 1^) was used when noted for easier visualization of sensitivity phenotypes and LB0N medium (tryptone 10 g l^− 1^, yeast extract 5 g l^− 1^, agar 16 g l^− 1^) was used to facilitate suppressor mutant formation. Ampicillin (200 µg ml^−1^ in LB; 100 µg ml^−1^ in M9) and kanamycin (50 µg ml^−1^) were used when appropriate. 4dPN toxicity assays were performed on thin (20 ml) media plates containing 0.02 % or 0.2 % arabinose and ampicillin when appropriate. For the purposes of this study LB or LB Lennox serve as rich medium and M9 serves as minimal medium.

As Δ*pdxA* and Δ*pdxB* Δ*pdxJ* (vitamin B_6_ auxotrophic mutants) are unable to grow in minimal medium alone, 4dPN sensitivity assays were carried out on M9 minimal medium plates supplemented with 1 µM and 5 µM each of PN and PL, unless otherwise specified.

Precultures of all strains were grown overnight at 37 °C, shaking in LB, with antibiotics when appropriate. For all physiological experiments, cells were diluted 1 : 500 from overnight cultures and grown at 37 °C, shaking in LB until early stationary growth had been reached (~ 6 h). Prior to experimental use, 1 ml cells were harvested by centrifugation, washed twice with and resuspended in 1X PBS solution (8 g l^−1^ NaCl, 0.2 g l^−1^ KCl, 1.88 g l^−1^ Na_2_HPO_4_⋅7H_2_O, 0.24 g l^−1^ KH_2_PO_4_; pH 7.4).

All media components were purchased from Fisher Scientific, apart from pyridoxine hydrochloride (PN) and pyridoxal hydrochloride (PL) which were purchased from Sigma-Aldrich and 4’-deoxypyridoxine hydrochloride (4dPN) that was purchased from Sigma-Aldrich and Cayman Chemical.

### Strain and plasmid constructions

All strains and plasmids used in this study are listed in Table S1 and all oligonucleotides in Table S2. The DNA encoding the *ygg*S gene was amplified by PCR with primers DH596 and DH598 from *E. coli* BW25113 genomic DNA and cloned in pBAD24 using restriction sites SphI and NcoI to give pBY291.3. pBAD24 and pBY291.3 constructs were verified by sequencing and transformed into the relevant strains for use in physiological studies. Electrocompetent cells were prepared, and all plasmid transformations were performed following standard procedures in 1 mm sterile electroporation cuvettes (Genesee Scientific) using a MicroPulser Electroporator (BioRad). Electroporation was performed using the preset bacterial protocol, resulting in consistent pulse duration of ~1.8 ms for each transformation. PLP-related mutants were constructed by P1 transduction utilizing the corresponding Keio collection strains, or NU812 (Table S1) in the case of Δ*pdxA::kan*, as donors [21]. All mutations were transduced into *Escherichia coli* str. K12 substr. BW25113 strain to ensure isogenic strain background and verified by PCR. Double and triple mutants were constructed by P1 transduction after eviction of the kanamycin resistance cassette [22]. P1 transductions were performed following standard procedures [23] and the sensitivity to P1 phage of all recipient strains used was verified.

### 4dPN sensitivity assays

For conducting the 4dPN sensitivity assays on solid media preparatory cultures were grown, and cells were harvested and washed as described in the ‘growth conditions and media’ section. Concentration of washed cells was normalized to an OD_600_ of 0.006 in 3 ml 1X PBS. Each strain was then plated on the media indicated in the results by sterile pouring of the normalized cell dilutions onto the plate, rotating to ensure complete coverage and pipetting to remove any excess liquid. Wells were made in the centre of each plate using the large end of sterile P200 pipet tips and plates were treated by adding 20 µl of 4dPN (25 mM concentration, unless otherwise specified) to the central well. Plates were incubated upright at 37 °C for 24 to 36 h, depending on the strain, prior to imaging. Plates were imaged using a CannonScann 5600F scanner. All experiments were repeated three times independently. Each figure represents plates from a single experiment with a single incubation length and plate images have been edited for easier visibility of phenotypes (sharpened by 50%, +20 % brightness, +20 % contrast in Microsoft PowerPoint v 16.63.1).

For conducting 4dPN sensitivity assays in liquid media, overnight cultures were washed and resuspended in physiological solution. Growth curves of the indicated *E. coli* strains were obtained by measuring the optical density at 600 nm with a Microplate Reader (ThermoScientific Multiskan GO). The *E. coli* strains were grown in rich or minimal media as indicated and supplemented with PL, PN or 4dPN at the indicated concentrations. Each curve represents the average of three independent experiments, each performed in triplicate and was fitted to a three-parameters growth equation using Prism (GraphPad):

$$Y=\frac{{OD}_{max}{\times OD}_{0}\times e^{rX}}{\left({OD}_{max}+{OD}_{0}\times e^{rX}\right)-1}$$

Where X is time, OD_0_ is the OD_600nm_ at time 0, OD_max_ is the maximal OD_600nm_ reached from the culture, and r is the rate of growth.

### Analysis of PN uptake by HPLC

An *E. coli* Δ*pdxK* Δ*pdxY* Δ*pdxI* derivative (JTB1590) in which the conversion of PN to alternative B_6_ vitamers is inhibited was used for conducting the PN uptake assays by HPLC. This strain was grown in 100 ml LB medium to exponential phase (≈ 4 h) at 37 °C. The bacterial pellet was washed three times with physiological solution and resuspended in 25 ml M9 +0.4 % glucose minimal medium. Then 1 ml aliquots of this resuspension were incubated at 30 °C for 5 min in the presence of PN and/or 4dPN. Uptake was stopped by rapid centrifugation and, after washing with M9 medium, the pellet was treated with 10 volumes of 0.7 M HClO_4_ and then with 0.7 M KOH. The mixture was centrifuged and the supernatant diluted in H_2_O was analysed by HPLC (Vanquish Core, ThermoFisher Scientific), equipped with both an UV (290 and 340 nm) and a fluorescence detector (excitation 290 nm, emission 395 nm). The separation was performed using a C18 Acclaim column (4.6×150 mm, particle size 5 µm, ThermoFisher Scientific) with 33 mM phosphoric acid and 8 mM 1-octanesulfonic acid, adjusted to pH 2.2 with KOH, as mobile phase A, and 80 % acetonitrile (vol/vol), as mobile phase B. The linear gradient was from 1 % mobile phase B to 2 % B for 5 min, 2 % B to 20 % B for 10 min, 20 % B to 30 % B for 5 min, and 30 % B to 90 % B for 5 min. A triplicate measurement was performed for each sample and the entire experiment was carried out three times.

### Tn-Seq and fitness assays

Fitness assays were run using the *E. coli* Keio_ML9 mutant library, a barcoded transposon (RB-Tn-Seq) library constructed in *E. coli Str. K12* BW25113, as described by Wetmore *et al*., 2015 [24]. Briefly, the Keio_ML9 library consists of a pool of many single-gene transposon mutants covering most of the *E. coli* genome. These transposon libraries can be used in competitive growth assays, allowing for analysis of fitness of the many *E. coli* mutants under a variety of growth conditions. An aliquot of the library was grown in 25 ml LB +33 µg ml^−1^ kanamycin, shaking at 37 °C until recovered to mid-log phase (~ 3.5 h). Cell pellets were collected to serve as a ‘time zero’ reference and the remaining cells were washed in 1 x PBS as described in the ‘growth conditions and media’ section, then normalized to an OD_600_ of 0.02 in 24-well microplates containing 1.2 ml of the indicated media and treatment conditions per well. Experimental plates were grown in a Multitron shaker at 37 °C. Four replicates were carried out for each control and experimental condition. Four experimental conditions were tested, all composed of M9 0.4 % glucose minimal media supplemented with the following concentrations of 4dPN/PN, respectively (ratio): 250 nM / 100 nM (2.5 ×), 25 µM / 5 µM (5 ×), 250 nM / 20 nM (12.5 ×), and 25 µM / 100 nM (250 ×). LB and M9 0.4 % glucose without supplemented PN or 4dPN served as control conditions. Barcode sequencing analysis was carried out as previously described [24] and gene fitness scores were analysed in the Fitness Browser (https://fit.genomics.lbl.gov/) [25].

### Isolation and identification of Δ*yggS* 4dPN-sensitivity suppressor mutants

For isolation of Δ*yggS* 4dPN resistant mutants, cells were plated on LB0N (LB, minus NaCl: tryptone 10 g l^− 1^, yeast extract 5 g l^− 1^, agar 16 g l^− 1^) as described in ‘4dPN sensitivity assays’. Plates were incubated upright at 37 °C for 24 h, then plates were placed upright on the benchtop for 48 more hours (72 h of growth total: 24 h at 37 °C, 48 h at room temperature) and 4dPN resistant clones were found to appear. Isolated resistant colonies were streaked from the area of growth inhibition onto selective medium (LB0*N*+5 µM 4dPN) and growth was compared to wild-type and parental Δ*yggS*. Many of these mutants exhibited inconsistent growth phenotypes over time, therefore several rounds of re-isolation were performed. Three Δ*yggS* derivatives (JTBS1, JTBS2, and JTBS3) were consistently able to grow on LB0*N*+5 µM 4dPN while the parental *yggS* mutant was not. These strains were streaked onto LB miniplates and sent for sequencing and variant calling (MiGS; Microbial Genome Sequencing Centre, LLC). Variant calling was performed by MiGS using BreSeq [26] and the complete *Escherichia coli* BW25113 genome assembly as a reference (GenBank: CP009273.1; RefSeq: NZ_CP009273.1).

### PdxK, PdxH and GlyA purifications and activity assays

PdxK, PdxH and GlyA were recombinantly expressed and purified to homogeneity as previously reported [27, 28].

Phosphorylation of 4dPN by PdxK was assayed using the ADP-Glo kinase assay kit (Promega), which allows the measurement of consumed ATP. Assays were performed in 20 mM KHEPES buffer pH 7.5, containing 0.5 mM MgCl_2_, 1 mM ATP, 1 mM PL/PN/4dPN and 0.25 µM PdxK, at 37 °C.

Kinetics of PL phosphorylation by PdxK were measured spectrophotometrically by measuring the absorbance at 390 nm of the produced PLP and using an extinction coefficient of 5330 M^−1^ cm^-1^ [29]. Reaction mixtures, in 20 mM KHEPES buffer pH 7.5 with 0.5 mM MgCl_2_, contained 1 mM MgATP, 0.1 mM PL and various concentrations of 4dPN (from 0 to 500 µM). Initial velocity measurements were carried out using the same method, with 1 mM MgATP and variable PL (from 6.25 to 400 µM) and 4dPN (from 0 to 25 µM).

Inhibition assays with PdxH were performed in 50 mM TRIS-HCl buffer pH 7.5 at 37 °C, using 1 µM PdxH, 1.2 µM PNP (which corresponds to Km for this substrate [29] and variable concentrations of 4dPNP (from 0 to 1.7 mM). The production of PLP as a function of time was measured spectrophotometrically at 420 nm, using an extinction coefficient of 4220 M^−1^ cm^−1^ [29].

4dPNP was obtained from 4dPN using PdxK (0.25 µM) as catalyst, in 20 mM KHEPES buffer pH 7.5 containing 0.5 mM MgCl_2_, 10 mM ATP, 10 mM 4dPN. After incubation for 16 h at 37 °C, the reaction mixture was filtered in a 10 kDa cut-off centrifuge filter to eliminate the enzyme. The concentration of produced 4dPNP was estimated from the concentration of ATP consumed in the reaction, which was measured using the ADP-Glo kinase assay kit (Promega). The effect of ADP present in the 4dPNP preparation (a stoichiometric amount with respect to 4dPNP) on PdxH activity was evaluated in an experiment in which 1.7 mM ADP was added in the reaction mixture used in the enzyme assay. No inhibition by ADP was observed.

GlyA activity measurements were carried out with 1 µM apoenzyme samples containing 1.2 µM PLP and different concentrations of PNP, 4dPN and 4dPNP. l-Serine and THF were used as substrates at 10 mM and 50 µM, respectively, in a spectrophotometric coupled assay, in which the 5, 10 methylene-THF produced in the SHMT reaction was oxidized by the NADP^+^-dependent *E. coli* 5, 10 methylene-THF [30].

## Results

### *Escherichia coli* cannot utilize 4dPN as a source of vitamin B_6_

It was previously reported that when present in high concentrations, 4dPN is utilized as a PLP precursor by an *E. coli* Δ*pdxH* mutant, a B_6_ auxotroph [19]. A commonly used method for synthesis of 4dPN is hydrogenation of PN, which can lead to 4dPN preparations that are contaminated with various species of B_6_ vitamers [31]. This leads to the question of whether 4dPN can truly act as a PLP precursor or if the previous finding was the result of contamination.

We found that the high concentration of 4dPN used in our plate sensitivity assays (25 mM) could not sustain growth of the Δ*pdxB* Δ*pdxJ* B_6_ auxotroph in minimal medium but the source of 4dPN was critical as some could be contaminated with alternate B_6_ vitamers (Fig. S1A). This led to our decision to use the compound purchased from Sigma-Aldrich and confirmed to be pure in all subsequent assays. Additionally, growth assays in liquid minimal medium confirmed that neither theΔ*pdxJ* nor Δ*pdxH* auxotrophic mutants were able to grow when supplemented with up to 10 mM 4dPN (Fig. S1B). Hence, even when present in very high concentrations, 4dPN cannot act as a source of vitamin B_6_ in *E. coli *.

### 4dPN sensitivity of wild-type *Escherichia coli* is dependent on medium composition

**Fig. 2.**
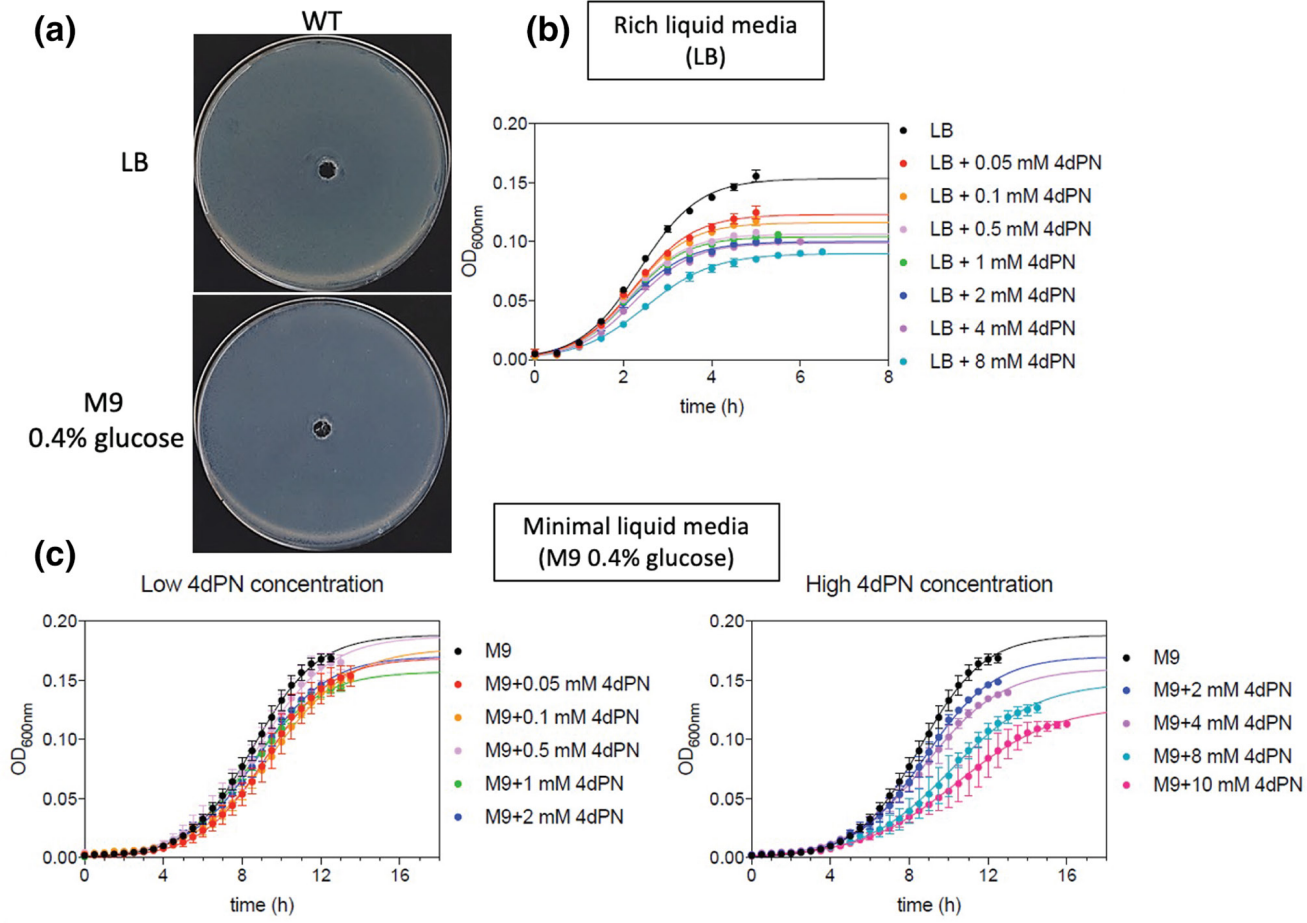
The 4-deoxypyridoxine sensitivity of wild-type *E. coli* is dependent upon media composition. (**a**) 4dPN sensitivity assays carried out on rich (LB) and minimal (M9 0.4 % glucose) solid media. Plated bacterial cells (OD_600_=0.006) were treated with 20 µl of 25 mM 4dPN in a central well, revealing no 4dPN sensitivity on solid media. All plate sensitivity experiments were repeated at least three times. (**b**) and (**c**) 4dPN sensitivity assays of the wild-type strain in liquid medium at the indicated concentrations. Each growth curve represents the average of three independent experiments, each performed in triplicate. The continuous line results from fitting to a three-parameters growth equation.

Plate sensitivity assays revealed little to no observed 4dPN toxicity in wild-type *E. coli* (BW25113) on either rich or minimal solid media treated with 25 mM 4dPN in a central well (Fig. 2a). However, when grown in liquid rich medium the wild-type strain exhibited a standard dose-dependent sensitivity response to 4dPN, with as little as 50 µM 4dPN inhibiting fully saturated growth and increasing concentrations leading to increasing growth inhibition, as exhibited by decreased optical density (Fig. 2b).

Wild-type *E. coli* also exhibited 4dPN sensitivity in liquid minimal medium, but interestingly this sensitivity was only detected when treated with a much higher concentration than required for observed sensitivity in rich medium (Fig. 2c). Additionally, experiments carried out comparing growth of mutants of PLP biosynthesis, salvage and PLP-dependent enzymes revealed that several mutants have growth characteristics that generally differ from wild-type and their 4dPN sensitivity also varied depending on medium composition. These results have been summarized in ‘Supplemental results’.

### 4dPN inhibits the growth of vitamin B_6_ auxotrophs

**Fig. 3.**
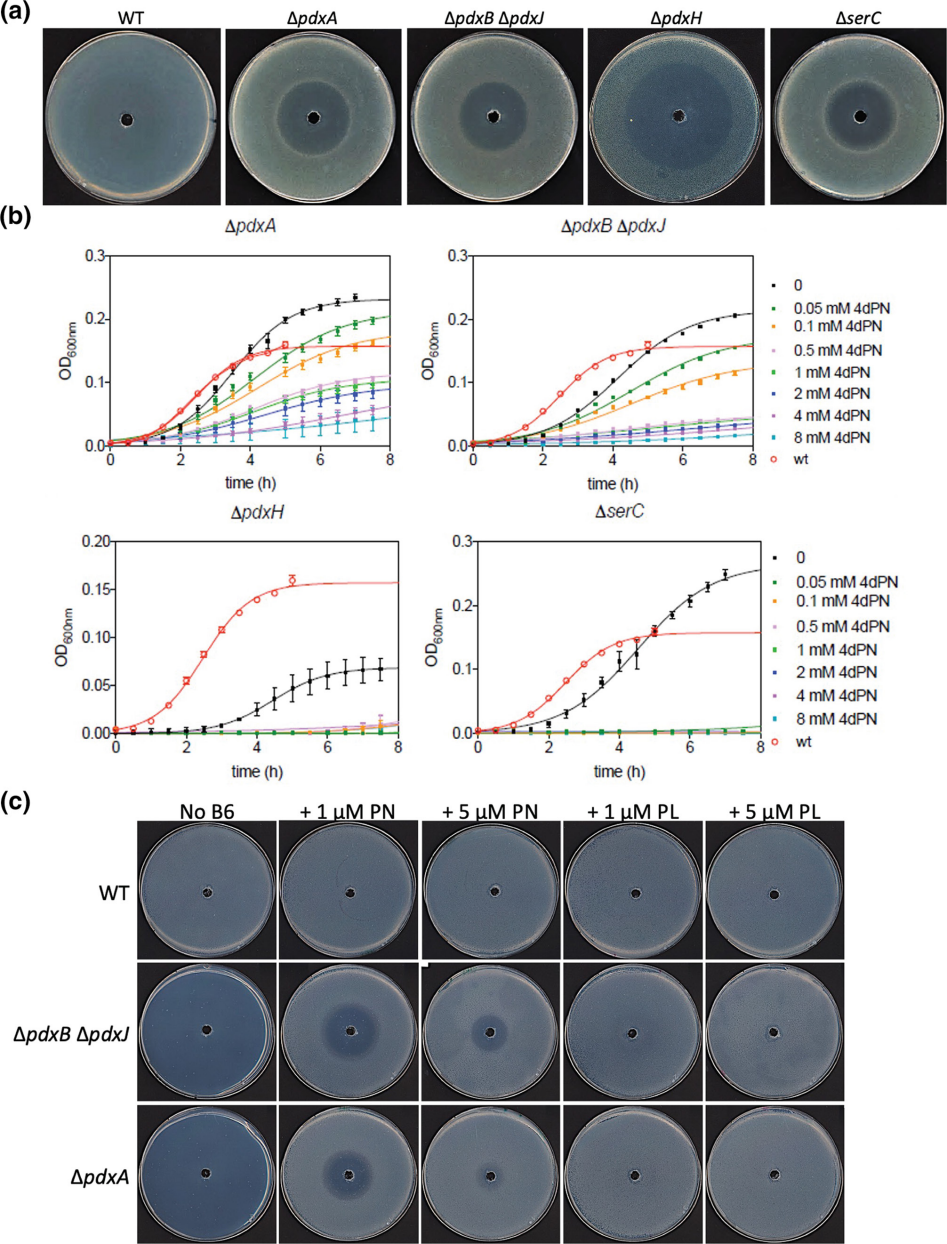
4-Deoxypyridoxine inhibits growth of *E. coli* vitamin B_6_ auxotrophs. (**a**) 4dPN sensitivity assays of Δ*pdxA*, Δ*pdxB*Δ*pdxJ*, Δ*pdxH*, and Δ*serC* mutants in rich (LB) solid media. Plated bacterial cells (OD_600_=0.006) were treated with 20 µl of 25 mM 4dPN in a central well. (**b**) Growth curves of the same mutants in liquid rich media treated with the specified range of 4dPN concentrations. Each growth curve represents the average of three independent experiments, each performed in triplicate. The continuous line results from fitting to a three-parameters growth equation. (**c**) 4dPN sensitivity assays of the WT, Δ*pdxB*Δ*pdxJ* and Δ*pdxA* strains on minimal medium plates supplemented with 1 µM or 5 µM of PN or PL and treated with 20 µl of 25 mM 4dPN in a central well. All plate sensitivity experiments were repeated at least three times.

To further characterize the physiology of 4dPN sensitivity, we first focused on mutants of the *de novo* PLP biosynthesis pathways (Fig. 1) that are auxotrophic for vitamin B_6_ (Δ*pdxA*, Δ*pdxB*Δ*pdxJ*, Δ*pdxH*, and Δ*serC*). The fact that a *pdxB* mutant has been shown to acquire suppressor mutations at a high rate allowing it to overcome its B_6_ auxotrophy [32] led us to use a Δ*pdxB*Δ*pdxJ* double mutant to represent both auxotrophs. 4dPN sensitivity assays on rich medium plates revealed that unlike wild-type *E. coli *, auxotrophic strains are highly sensitive to 4dPN, as treatment in a central well resulted in a large, concentration-dependent central zone of growth inhibition (Fig. 3a). This phenotype was also observed in liquid rich medium for Δ*pdxA* and Δ*pdxB*Δ*pdxJ* mutants, where growth inhibition was observed with as little as 50 µM 4dPN and increased with higher 4dPN concentrations (Fig. 3b). The responses of Δ*pdxH* (which is a mutant of both PLP biosynthesis and salvage) and Δ*serC* (which is a mutant of both PLP and serine biosynthesis and auxotrophic for both B_6_ and l-serine) to 4dPN treatment in liquid rich medium were more severe, with all concentrations of 4dPN tested leading to complete growth inhibition. Additionally, Δ*pdxH* is known to have generally defective growth [33]. In agreement, our assays show that growth of Δ*pdxH* is reduced in all media tested, resulting in a much lower final cell density than wild-type or the other auxotrophs (Fig. 3b and Supplemental results).

As the auxotrophic strains are unable to grow in minimal medium alone, sensitivity assays for Δ*pdxA* and Δ*pdxB*Δ*pdxJ* were carried out on minimal medium plates supplemented with PN or PL. Due to their complex phenotypes and involvement in multiple metabolic pathways, 4dPN toxicity assays in minimal medium were not performed for Δ*pdxH* and Δ*serC* mutants.

Similar to rich medium, the B_6_ auxotrophs exhibit a sensitivity phenotype on minimal media supplemented with B_6_ that was visualized as a zone of growth inhibition, but this phenotype was influenced by both the concentration and identity of the supplemented vitamer (Fig. 3c). By increasing the concentration of supplemented PN from 1 µM to 5 µM the zone of growth inhibition decreases in diameter, suggesting that in agreement with previous findings, 4dPN may competitively inhibit the uptake of exogenous PN by vitamin B_6_ auxotrophs [19]. Additionally, no 4dPN sensitivity was seen in either mutant on minimal medium supplemented with PL, suggesting that 4dPN inhibits uptake of PN specifically (Fig. 3c).

Further analyses confirmed that the Δ*pdxB*Δ*pdxJ* sensitivity to 4dPN on minimal medium plates was dependent on the concentrations of both PN and 4dPN. As shown in Fig. S2, the greatest ratio of 4dPN to PN (treatment with 20 µl of 25 mM 4dPN diffused from the central well of plates supplemented with 500 nM PN) resulted in the largest zone of growth inhibition, while the lowest ratio of 4dPN to PN (treatment with 20 µl of 6.25 mM 4dPN diffused from a central well of plates supplemented with 5 µM PN) essentially eliminated the 4dPN sensitivity of the auxotroph.

### 4dPN sensitivity of vitamin B_6_ auxotrophs is due to inhibition of B_6_ phosphorylation, resulting in loss of cumulative uptake

**Fig. 4.**
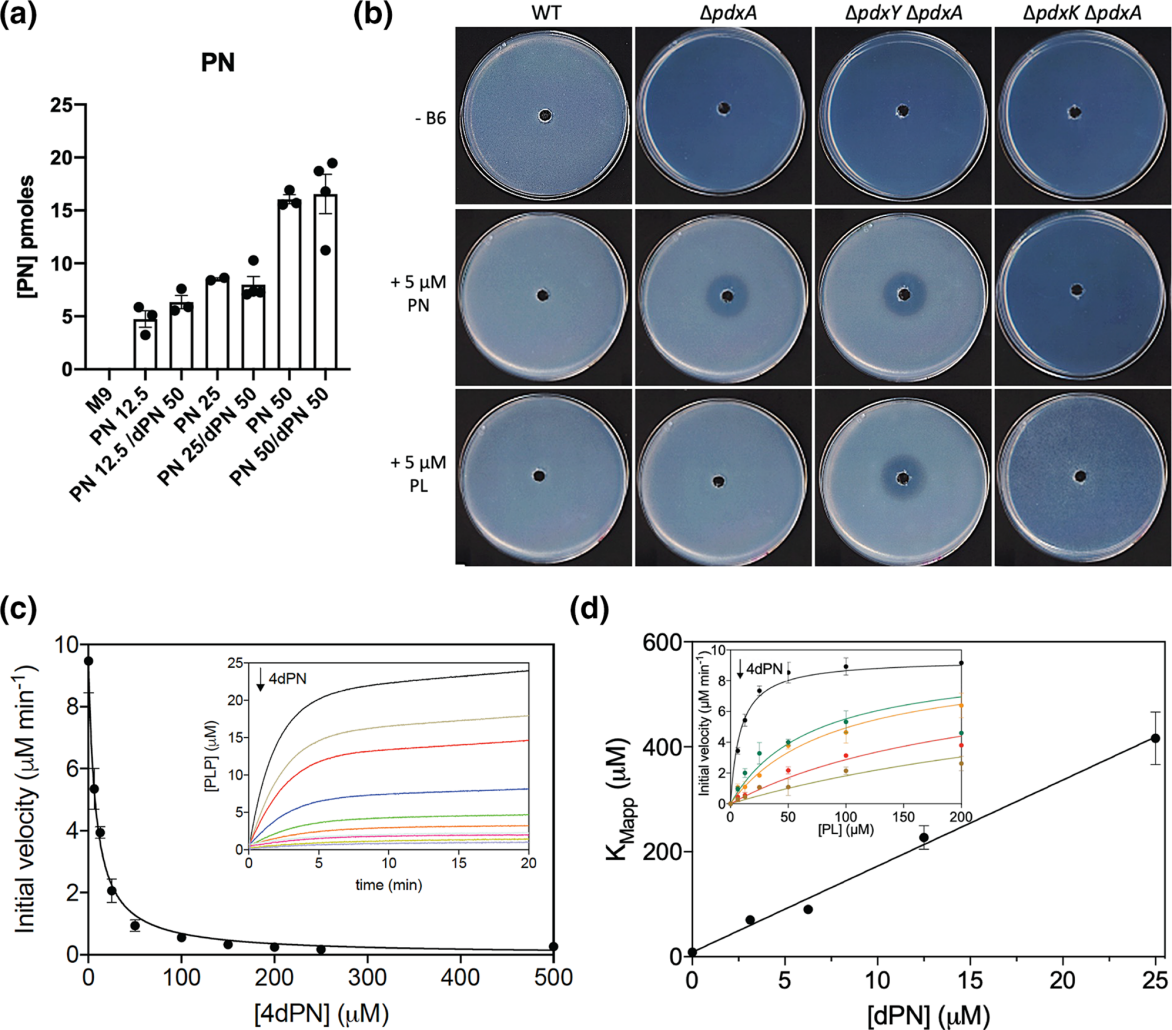
4-Deoxypyridoxine sensitivity of vitamin B_6_ auxotrophs is due to inhibition of B_6_ phosphorylation, resulting is loss of cumulative uptake. (**a**) Analysis by HPLC of PN uptake in the *E. coli* Δ*pdxK* Δ*pdxY* Δ*pdxI* strain. Bacterial cells were incubated in M9 in the presence of 12.5 µM PN, 12.5 µM PN +50 µM 4dPN, 25 µM PN, 25 µM PN +50 µM 4dPN, 50 µM PN, and 50 µM PN +50 µM 4dPN (see Methods section for details). Internalized PN and 4dPN were separated and measured by HPLC as described in Methods. (**b**) 4dPN sensitivity assays of the WT, Δ*pdxA,* Δ*pdxK,* Δ*pdxY,* Δ*pdxK*Δ*pdxA* and Δ*pdxY pdxA* strains on minimal medium plates supplemented with 1 µM or 5 µM of PN or PL. Plated bacterial cells (OD_600_=0.006) were treated with 20 µl of 25 mM 4dPN in a central well. All plate sensitivity experiments were repeated at least three times. (**c**) 4dPN inhibits PdxK. The conversion of PL into PLP catalysed by PdxK was measured in the presence of increasing concentrations of 4dPN. Reaction mixtures, in 20 mM KHEPES buffer pH 7.5 at 37 °C with 0.5 mM MgCl_2_, contained 1 mM MgATP, 0.1 mM PL and various concentrations of 4dPN (from 0 to 500 µM). The figure shows the dependence of the initial velocity of the reactions (measured as the slope of the initial linear production of PLP; inset) as a function of 4dPN. (**d**) 4dPN inhibition of PdxK is competitive. Saturation curves (inset) were obtained by varying PL concentration (from 6.25 to 400 µM), while keeping MgATP concentration fixed at 1 mM, at different 4dPN concentrations (from 0 to 25 µM). Saturation curves were globally fitted using the Michaelis-Menten equation, with the V_max_ as shared parameter. The figure shows that the apparent K_M_ obtained by the fitting procedure varies linearly as a function of 4dPN concentration, indicating a competitive inhibition. An inhibition constant of about 0.5 µM was estimated by the intercept of the continuous straight line, obtained through the linear regression of data, on the X-axes.

Cumulative uptake of extracellular B_6_ requires transport of non-phosphorylated vitamers followed by rapid phosphorylation by B_6_ kinases to effectively ‘trap’ the vitamers [12]. Therefore, the competitive inhibition of PN uptake by 4dPN resulting in the auxotrophic sensitivity phenotype could be caused by either inhibition of PN transport at the membrane or through inhibition of phosphorylation resulting in loss of cumulative uptake. To test these hypotheses, first a strain that lacked the ability to convert PN to alternative vitamer forms (Δ*pdxK*Δ*pdxY*Δ*pdxI*) was constructed and intracellular PN concentrations were measured when this mutant was grown in minimal medium supplemented with PN, with and without various concentrations of 4dPN treatment. The presence of 4dPN did not affect the uptake of extracellular PN (Fig. 4a), therefore 4dPN sensitivity of the auxotrophic strains cannot be through competitive inhibition of PN membrane transport, directly.

Alternatively, if 4dPN competes with PN for PdxK-specific activity, PN would be able to be transported across the membrane, but cumulative uptake would decrease through competitive inhibition of PdxK by 4dPN. The presence of a second, PL-specific B_6_ kinase in *E. coli *, PdxY, would explain the lack of 4dPN sensitivity of auxotrophic strains grown in the presence of PL, which can still be phosphorylated to PLP.

To test this hypothesis, auxotrophic strains deficient in PL- and B_6_ kinase activity (Δ*pdxY*Δ*pdxA* and Δ*pdxK*Δ*pdxA*, respectively) were constructed and plate 4dPN sensitivity assays were carried out. Our results showed that 4dPN sensitivity of the PL-kinase deficient auxotroph (Δ*pdxY*Δ*pdxA*) on minimal medium supplemented with PL was nearly identical to that of the auxotroph (Δ*pdxA*) on minimal medium supplemented with PN (Fig. 4b). This finding confirms that inhibition of cumulative PN uptake by 4dPN is through inhibition of phosphorylation. Additionally, the loss of PL-kinase activity had no effect on 4dPN sensitivity when supplemented with PN, suggesting that inhibition of B_6_ kinase activity by 4dPN is PdxK-specific. Further, deletion of *pdxK* in the auxotrophic background resulted in a slower growth rate and an inability for the mutant to utilize PN but did not lead to 4dPN sensitivity when supplemented with PL (Fig. 4b).

Activity assays confirmed that, *in vitro*, PdxK is effectively inhibited by 4dPN in a concentration-dependent manner, so that the initial velocity of PL phosphorylation catalysed by the enzyme decreases hyperbolically as 4dPN is increased (Fig. 4c). A more detailed kinetic analysis showed that 4dPN inhibition is indeed of a competitive nature, which allowed the determination of an inhibition constant of 0.5±0.6 µM (Fig. 4d). Compared to the K_m_ value for PN, which from the same data is estimated as 8.6±1.2 µM, the lower value of this inhibition constant indicates that 4dPN is actually a better substrate than PN. These results show that while PdxK is competitively inhibited by 4dPN, this competition exists between not only PN but PL as well. Therefore, PdxY is likely not inhibited by 4dPN and PdxY activity is enough to overcome 4dPN sensitivity of the auxotrophs in the presence of PL.

### The absence of YggS causes an increased and concentration-specific sensitivity to 4dPN

**Fig. 5.**
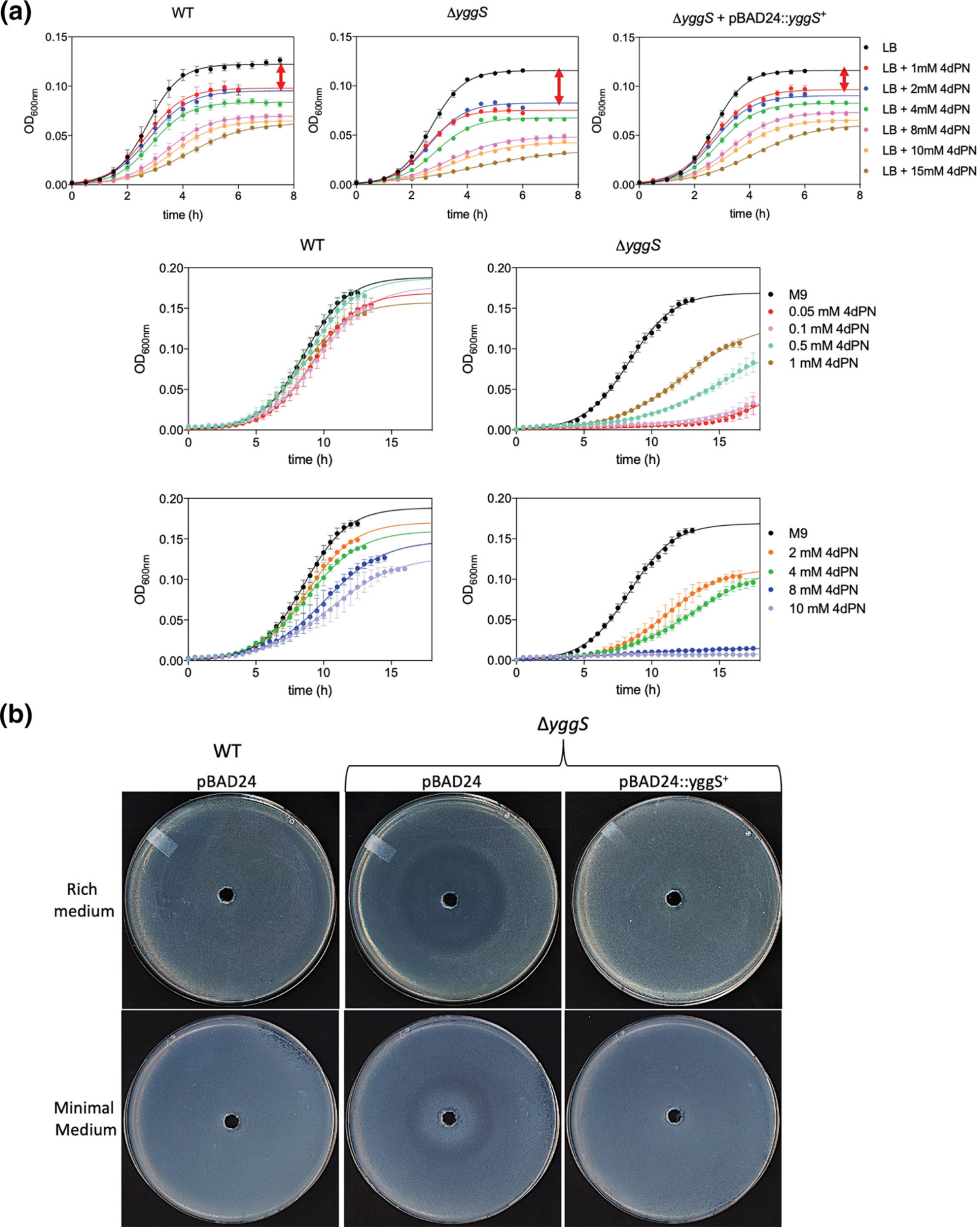
The absence of YggS leads to 4-deoxypyridoxine sensitivity in both rich and minimal media. (**a**) Growth curves of Δ*yggS* and WT strains carrying the pBAD24 control or the *yggS* expressing plasmid (pBAD::*yggS* ^+^) in both rich (LB) and minimal (M9) media treated with the specified range of 4dPN concentrations. (**b**) 4dPN sensitivity plates assays of the same strains in both rich (LB Amp 200 µg ml^−1^) and minimal (M9 0.4 % glucose Amp 100 µg ml^−1^ +0.02 % arabinose) media. Plated bacterial cells (OD_600_=0.006) were treated with 20 µl of 25 mM 4dPN in a central well. All plate sensitivity experiments were repeated at least three times. Each growth curve represents the average of three independent experiments, each performed in triplicate. The continuous line results from fitting to a three-parameters growth equation.

Previous studies have shown that vitamin B_6_ homeostasis is disrupted in *E. coli* Δ*yggS* [4, 34] and we used treatment with 4dPN to further perturb this homeostasis defect. 4dPN sensitivity assays revealed that the *yggS* mutant is more sensitive to 4dPN than the wild-type strain in both minimal and rich media (Fig. 5). In rich liquid medium, like wild-type, the Δ*yggS* strain shows a standard concentration-dependent sensitivity response to 4dPN, with increasing concentrations of 4dPN leading to increasing growth inhibition (Fig. 5a, top panels). Unlike in rich medium, where Δ*yggS* is only slightly more sensitive than wild-type, its sensitivity response in liquid minimal medium is much more pronounced and follows a unique profile, with both the highest and lowest concentrations of 4dPN leading to the most growth inhibition (Fig. 5a, middle and bottom panels).

On rich medium plates, the *yggS* mutant exhibits a clear sensitivity to 4dPN not seen in wild-type (Fig. 5b). This sensitivity phenotype differs from that seen in the auxotrophic strains and is similar to the PN sensitivity phenotype of Δ*yggS*, which can be seen as a concentration-dependent ring, rather than central zone, of growth inhibition [4]. On minimal medium plates, the *yggS* mutant shows a slight sensitivity to 4dPN with a ring of sparse growth, but this phenotype is less dramatic than the sensitivity exhibited in minimal liquid or rich solid media (Fig. 5b). The differences we see in the Δ*yggS* 4dPN sensitivity phenotypes between liquid and solid media are intriguing and not yet understood. The phenotypes are fully complemented by expressing the wild-type *yggS* gene *in trans* in both liquid and solid media (Fig. 5). Cells growing inside the ring of growth inhibition were isolated and retested for 4dPN sensitivity. As seen in Fig. S3 the ring phenotype was repeated, confirming that this unusual phenotype is due to a true physiological effect and does not simply result from acquired suppressor mutations.

### The 4dPN toxicity phenotype of a *yggS* mutant is influenced by the presence of B_6_ vitamers

**Fig. 6.**
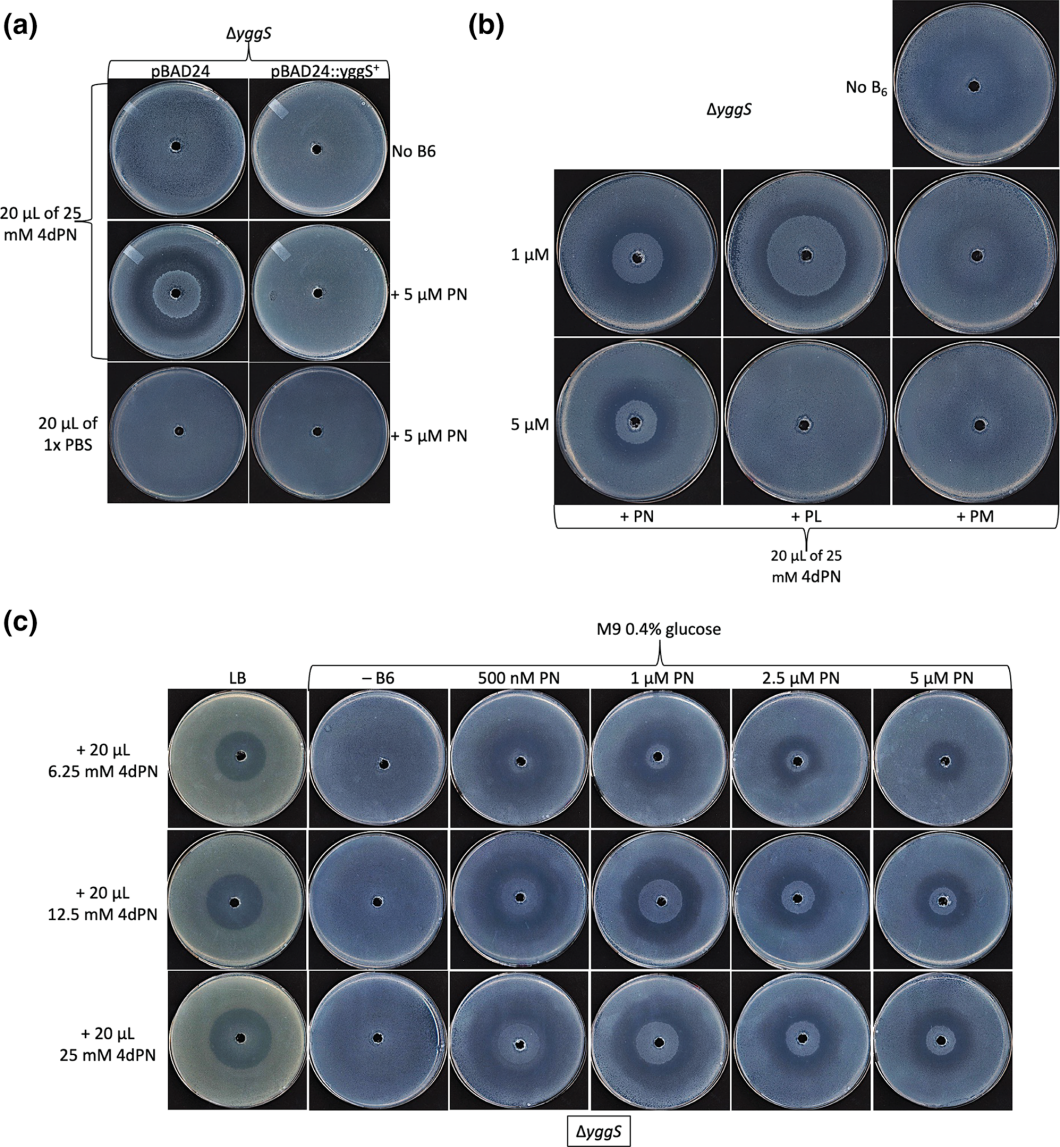
The 4-deoxypyridoxine sensitivity phenotype is influenced by the presence of B_6_ vitamers. (**a**) 4dPN sensitivity assays of Δ*yggS* and WT strains carrying the pBAD24 control or the *yggS* expressing plasmid (pBAD::*yggS* ^+^) on minimal medium plates (M9 0.4 % glucose Amp 100 µg ml^−1^ +0.02 % arabinose) with or without 5 µM PN. Plated bacterial cells (OD_600_=0.006) were treated with 20 µl of either 25 mM 4dPN or PBS (control) in a central well (**b**) 4dPN sensitivity assays ofΔ*yggS* on minimal medium plates supplemented with either 1 µM or 5 µM exogenous B_6_ vitamers. Plated bacterial cells (OD_600_=0.006) were treated with 20 µl of 25 mM 4dPN in the centre well, showing a distinct change in sensitivity depending on both the concentration and identity of the vitamer. (**c**) 4dPN sensitivity assays of Δ*yggS* on minimal medium plates supplemented with the indicated concentrations of PN and treated with 20 µl of either 6.25 mM, 12.5 mM or 25 mM 4dPN. All plate sensitivity experiments were repeated at least three times.

Given that 4dPN and PN compete for cumulative uptake, we predicted that the slight 4dPN sensitivity phenotype of Δ*yggS* on minimal medium plates would be suppressed by supplemented PN. To our surprise, the 4dPN sensitivity phenotype was greatly exacerbated by the presence of exogenous PN on minimal medium plates (Fig. 6a). Additional assays revealed that the severity of the 4dPN sensitivity phenotype exhibited by Δ*yggS* is also influenced by the presence of PL or PM but varied with the identity and concentration of the supplemented B_6_ vitamer (Fig. 6b). While a 4dPN sensitivity phenotype is observed in minimal medium supplemented with both high (5 µM) and low (1 µM) concentrations of PN, plates with 1 µM PN showed a larger, more pronounced ring of toxicity than those without B_6_ or high concentrations of PN (Fig. 6b). Little to no 4dPN sensitivity is seen on minimal media plates supplemented with high concentrations of PL or PM. However, on plates supplemented with only 1 µM PL, the *yggS* mutant exhibits a ring of 4dPN toxicity while the presence of 1 µM PM led to a smaller, but observable ring of defective growth similar to the phenotype seen without B_6_ (Fig. 6b).

Further exploration of the effect of 4dPN (treatment) to PN (supplemented in plates) ratios was carried out using a broader range of both PN and 4dPN concentrations. The results confirm that while the ratio of 4dPN to PN appears to be important and have an influence on 4dPN sensitivity of Δ*yggS*, this sensitivity does not follow a standard concentration-dependent pattern (Fig. 6c).

### Tn-Seq data confirms the fitness defect in a *yggS* mutant during PN, 4dPN growth challenges

The 4dPN sensitivity phenotype of the *yggS* mutant was further confirmed in a genome-wide mutant fitness experiment analysing the fitness consequences of growth in liquid minimal medium supplemented with PN and 4dPN on a barcoded transposon mutant library (RB-Tn-Seq) of *E. coli* BW25113. Disruption of YggS activity leads to a major fitness defect when both PN and 4dPN are present in the growth medium (Data S1). The Fitness Browser database uses data from RB-Tn-Seq experiments to provide genome-wide log2 fold fitness scores for transposon-insertion mutants under different experimental conditions [25]. Published fitness scores are available for a *yggS* mutant in 168 experiments but only eight conditions gave strong fitness scores (|fitness|>2 and |t|>5). Strong fitness defect scores (<−2) were derived from only three conditions. Further, fitness defect scores for a *yggS* mutant resulting from the PN/4dPN challenge experiments in this study were greater than any other published condition (Table 1). Among hundreds of unpublished experiments with data in the Fitness Browser database, a strong phenotype is rarely seen for *yggS* (data not shown).

**Table 1.** Fitness browser: conditions leading to strong fitness defects in an *Escherichia coli* BW25113 Δ*yggS* mutant

Experimental condition	Fitness score	t-value
M9 0.4 % glucose 2.5×4dPN/PN challenge (250 nM/100 nM)	−6.23	−6.06
M9 0.4 % glucose 250×4dPN/PN challenge (25 µM/100 nM)	−6.11	−5.95
M9 0.4 % glucose 12.5×4dPN/PN challenge (250 nM/20 nM)	−5.84	−6.94
M9 0.4 % glucose 5×4dPN/PN challenge (25 µM/5 µM)	−4.96	−7.56
Outer cut, LB soft agar motility assay	−4.9	−5.1
2-Furfuraldehyde (0.25 vol%)	−3.0	−3.5
l-Malic acid (carbon source)	−2.0	−3.8

*For conditions with multiple strong fitness defect scores, the strongest score and relevant t-value statistic are shown* https://fit.genomics.lbl.gov/cgi-bin/singleFit.cgi?orgId=Keio&locusId=17030&showAll=0

4dPN, 4'-deoxypyridoxine; PN, pyridoxine.

The experiments were carried out by supplementing minimal medium with four ratios of excess 4dPN to PN; 2.5 ×, 5 ×, 12.5 × and 250 ×. Regardless of the PN and 4dPN concentrations used in each challenge, a *yggS* mutant consistently showed a strong fitness decrease when both compounds were present. Importantly, the mutant exhibited a drastic fitness defect in M9 treated with 4dPN/PN compared to M9 alone (average M9 fitness scores are nearly nine times higher than average fitness scores in M9 supplemented with PN/4dPN) and no significant phenotype was seen in LB (Data S1).

In agreement with the plate and liquid 4dPN sensitivity assays described above, the Tn-seq data shows that the *yggS* mutant doesn’t exhibit a standard concentration-dependent sensitivity phenotype, as the lowest and highest ratios of excess 4dPN to PN lead to the greatest growth defects, with intermediate ratios leading to slightly less defective growth (Table 1, Supplemental results).

### The phosphorylated form of 4dPN is responsible for the toxicity phenotype in Δ*yggS*

**Fig. 7.**
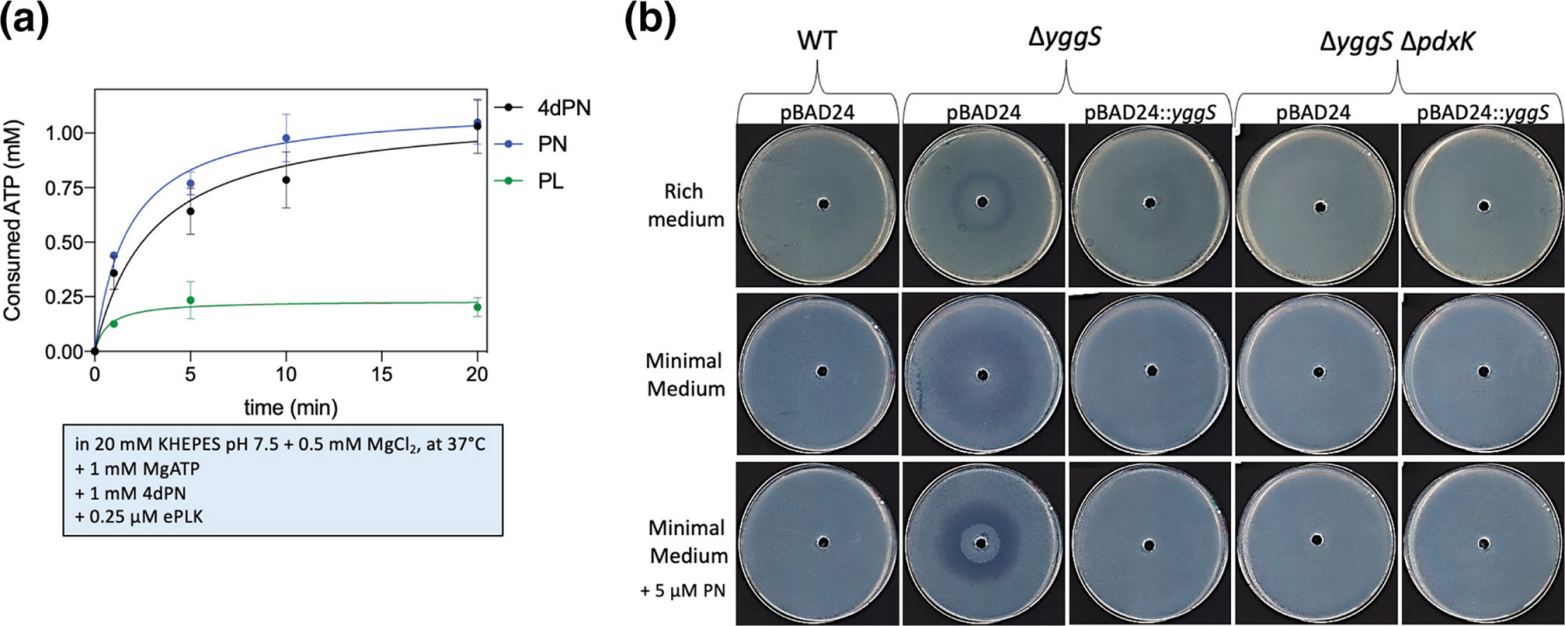
Phosphorylation of 4-deoxypyridoxine catalysed by PdxK and results in the visualized sensitivity of Δ*yggS*. (**a**) Phosphorylation reactions were carried out as described in Methods (reactions were performed in 20 mM KHEPES buffer pH 7.5 containing 0.5 mM MgCl_2_, 1 mM ATP, 1 mM PL/PN/4dPN and 0.25 µM PdxK, at 37 °C.) to assess the ability of PdxK to phosphorylate 4dPN and followed by measuring the consumed ATP, using the ADP-Glo kinase assay kit (Promega). (**b**) 4dPN sensitivity assays of Δ*yggS* and Δ*yggS* Δ*pdxK* mutants carrying the pBAD24 control or the *yggS* expressing plasmid (pBAD::*yggS* ^+^) on rich (LB Amp 200 µg ml^−1^ +0.02 % arabinose) and minimal (M9 0.4 % glucose Amp 100 µg ml^−1^ +0.02 % arabinose, with or without 5 µM PN supplemented) media. Plated bacterial cells (OD_600_=0.006) were treated with 20 µl of 25 mM 4dPN in the centre well. All plate sensitivity experiments were repeated at least three times.

A *yggS* mutant accumulates higher levels of intracellular PNP with respect to wild-type, which has been shown to inhibit PLP-dependent enzymes, such as that of the glycine cleavage system [18]. Our previous results indicate that 4dPN appears to compete with PN for phosphorylation (Fig. 4). As 4dPN is a PN analogue, we hypothesized that like PN, 4dPN is likely also rapidly phosphorylated by PdxK and previous studies showed that the PdxK homolog from chick embryo has a high affinity for 4dPN [35]. Indeed*, in vitro* activity assays on recombinantly expressed and purified *E. coli* PdxK showed that it phosphorylates 4dPN using ATP (Fig. 7a).

*In vivo* studies were carried out to investigate if the activity of PdxK was required for the observed 4dPN sensitivity phenotype in Δ*yggS*. 4dPN sensitivity assays were performed in a Δ*yggS*Δ*pdxK* double mutant strain, revealing that deletion of *pdxK* suppressed the 4dPN sensitivity phenotype of the *yggS* mutant (Fig. 7b). This finding confirms the PdxK 4dPN kinase activity *in vivo* and indicates that 4dPNP is likely the source of the observed 4dPN toxicity.

The Δ*yggS* strain develops a high rate of suppressor mutations when treated with 4dPN on LB0N plates (LB, minus NaCl). To further investigate the severe sensitivity of the *yggS* mutant to 4dPN, three stable Δ*yggS* 4dPN-resistant derivatives were isolated and sequenced for identification of potential suppressor mutations (variant calling): JTBS1, JTBS2 and JTBS3 (see Methods). Sequences of the three isolates were compared to the wild-type *E. coli* BW25113 parent strain. In addition to missing coverage of the *yggS* gene region, each mutant contained the same three mutations, including a 39 base pair deletion in the *pdxK* coding region (Data S2). This supports our findings that PdxK activity is required for 4dPN toxicity and that loss of PdxK activity is beneficial during growth in the presence of 4dPN.

### Exacerbation of the *yggS* mutant 4dPN sensitivity phenotype by PN requires accumulation of both phosphorylated vitamers

**Fig. 8.**
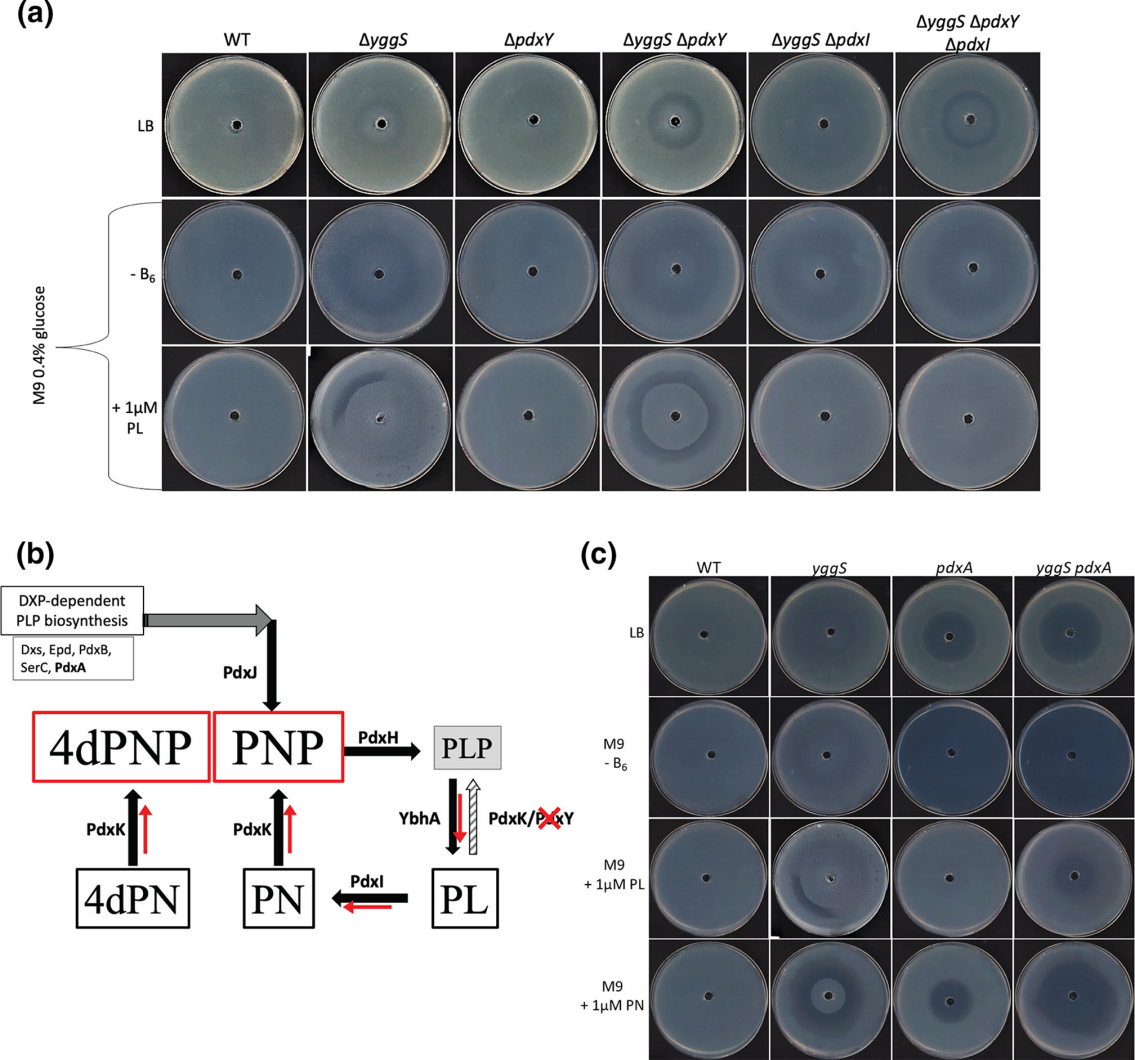
The exacerbated 4-deoxypyridoxine sensitivity phenotype of Δ*yggS* is the result of accumulation of both 4-deoxypyridoxine-phosphate and pyridoxine-phosphate. (**a**) 4dPN sensitivity assays on rich medium plates and minimal medium plates with or without 1 µM PL of WT, *yggS*, *pdxY*, *yggS pdxY*, *yggS pdxI* and *yggS pdxY pdxI* strains. Plated bacterial cells (OD_600_=0.006) were treated with 20 µl of 25 mM 4dPN in a central well. (**b**) Overview of vitamin B_6_ salvage in *Escherichia coli *. The red arrows and boxes indicate the hypothesized metabolic flux of B_6_ vitamer concentrations in a Δ*yggS* strain when *pdxY* is deleted (red cross) and/or PN and 4dPN are given exogenously, resulting in a build-up of 4dPNP and PNP. The gray-striped arrow indicates a reduced flux from PL to PLP when *pdxY* is deleted. (**c**) 4dPN sensitivity of the Δ*yggS*, Δ*pdxA*, and Δ*yggS*Δ*pdxA* mutants on rich medium and minimal medium with and without supplemented 1 µM PL and PN. Plated bacterial cells (OD_600_=0.006) were treated with 20 µl of 25 mM 4dPN in a central well. All plate sensitivity experiments were repeated at least three times.

One particularly interesting finding was that both *yggS* and *pdxY* mutants exhibit concentration dependent rings, rather than zones, of growth inhibition during 4dPN sensitivity assays on rich media plates with low salt concentrations (LB Lennox) (Supplemental results). Further investigations revealed that a Δ*yggS*Δ*pdxY* double mutant showed an exacerbated 4dPN sensitivity phenotype than either mutant alone on both rich medium and minimal medium supplemented with PL (Fig. 8a). Unlike Δ*yggS*, Δ*pdxY* is not sensitive to 4dPN on minimal medium plates supplemented with PL (Fig. 8a), hence this phenotype is most certainly not a direct effect of accumulation of high intracellular PL levels resulting from the loss of PdxY activity. With an increased abundance of PL in the Δ*yggS* Δ*pdxY* mutant, PdxI activity would likely convert the high levels of PL to PN, which would then be further converted to PNP via PdxK (Fig. 8b), ultimately resulting in the well-established PNP toxicity phenotype observed in a Δ*yggS* strain [4, 18]. Indeed, as shown Fig. 8a, deletion of *pdxI* in both the Δ*yggS* single and Δ*yggS*Δ*pdxY* double mutant backgrounds partially suppressed the 4dPN sensitivity phenotype on rich medium and fully suppressed the pronounced 4dPN sensitivity ring phenotype seen on minimal medium supplemented with PL. As the Δ*yggS*Δ*pdxY*Δ*pdxI* triple mutant is not sensitive to 4dPN on minimal medium with PL, the clear 4dPN ring sensitivity phenotype observed on rich medium is most certainly caused by the presence of PN in the medium (Fig. 8a). Deletion of *pdxY* and/or *pdxI* in the Δ*yggS* mutant background does not influence 4dPN sensitivity on minimal medium supplemented with PN (Fig. S4).

PdxA activity is required for synthesis of PNP prior to oxidation to PLP via the *de novo* synthesis pathway (Fig. 8b) and like *pdxY* and *pdxI* mutants, Δ*pdxA* exhibits no 4dPN sensitivity on minimal media supplemented with PL (Fig. 8c and Fig. 3c). Surprisingly, 4dPN sensitivity assays revealed that a *yggS pdxA* double mutant, with decreased *de novo* PNP production, has a very different sensitivity profile from the single *yggS* mutant (Fig. 8c). When plated on minimal medium supplemented with PL, Δ*yggS* exhibits the expected ring of sensitivity, but the Δ*yggS* Δ*pdxA* mutant loses this *yggS*-related ring phenotype and instead exhibits a small central zone of growth inhibition. As Δ*yggS* Δ*pdxA* should have decreased levels of intracellular PNP, this finding suggests that a *yggS* mutant is much less sensitive to 4dPN when there is less accumulated PNP in the cell. Alternatively, there is a visible increase in 4dPN sensitivity in the Δ*yggS* Δ*pdxA* mutant versus Δ*yggS* on both rich medium and minimal medium plates supplemented with PN. Δ*yggS* Δ*pdxA* exhibits a central zone of inhibition which could result from the inhibition of cumulative PN uptake (B_6_ availability) in the auxotrophic strain, but the expanded area of growth inhibition was unexpected (Fig. 8c).

### Both 4dPN and 4dPNP competitively inhibit important PLP/PNP binding enzymes

**Fig. 9.**
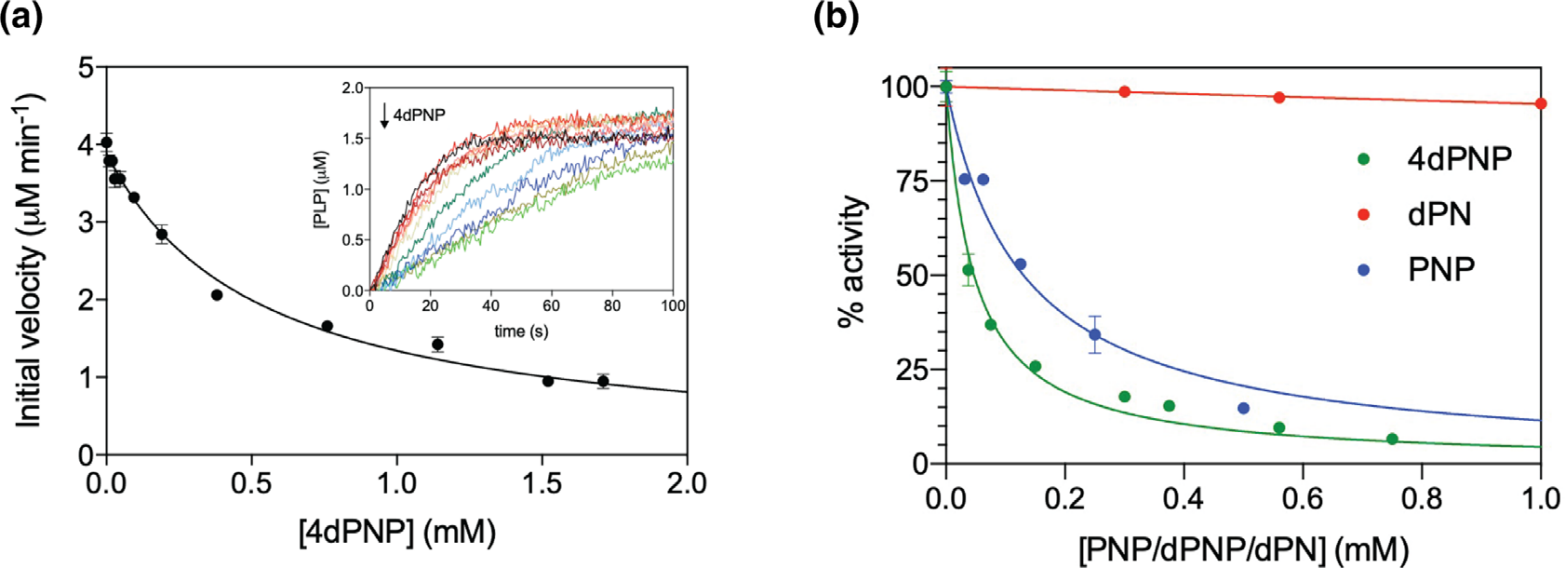
Inhibition of PdxH and GlyA by 4dPN, 4dPNP, and PNP. (**a**) 4dPNP inhibits PdxH. The kinetics of PNP conversion into PLP catalysed by PdxH was measured at increasing 4dPNP concentrations (from 0 to 1.7 mM) in 50 mM TRIS-HCl buffer pH 7.5 at 37 °C, using 1 µM PdxH and 1.2 µM PNP (inset). The figure shows how the initial velocity of the reaction as a function of 4dPNP concentration decreases hyperbolically. (**b**) 4dPNP and PNP inhibit GlyA activity. GlyA activity was measured after 15 min incubation of 1 µM apoenzyme samples containing 1.2 µM PLP and different concentrations of PNP, 4dPN and 4dPNP in 20 mM potassium phosphate buffer pH 7.2 at 30 °C. Activity decreased hyperbolically as a function of PNP and 4dPN concentration.

We have shown that 4dPN competitively inhibits PdxK, and previous studies suggest that 4dPNP is likely to competitively inhibit PdxH, another PLP salvage enzyme [36]. Activity assays performed using recombinantly expressed and purified PdxH, confirmed that PdxH is indeed inhibited by 4dPNP in a concentration-dependent manner (Fig. 9a), with an apparent inhibition constant of 0.53±0.03 mM.

GlyA is a highly prevalent, multifunctional PLP-dependent enzyme that plays a key role in *E. coli* metabolism through synthesis of 5,10-methylene-tetrahydrofolate, the major C1 unit provider of the cell [37]. *In vitro* analysis of the effects of 4dPN, PNP, and 4dPNP treatments on GlyA were carried out. While the presence of 4dPN did not affect GlyA activity, the presence of either PNP or 4dPNP was strongly inhibitory (Fig. 9b; apparent inhibition constants equal to 0.05±0.03 mM for 4dPNP and 0.13±0.01 mM for PNP).

## Discussion

Vitamin B_6_ is essential for life and through its role as a cofactor has a wide-reaching influence on general metabolism and physiology in *E. coli *. The presence of an anti-vitamin B_6_ metabolite disrupts the balance of typical cellular B_6_ vitamers ratios and due to the importance of PLP, leads to a range of pleiotropic phenotypes. In this study we characterized the sensitivity of *E. coli* to 4dPN, a PN analogue and vitamin B_6_ antimetabolite, in both wild-type and PLP biosynthesis, salvage and homeostasis mutant strains.

Importantly, 4dPN-induced growth defects can be identified in wild-type, suggesting that *E. coli* has not evolved a specific mechanism in which to differentiate 4dPN from other B_6_ vitamers nor detoxify the anti-metabolite when present in high concentrations within the cell. Interestingly, although the toxic effects of 4dPN are seen in both rich and minimal liquid media, a much higher concentration of 4dPN is required to detect sensitivity in minimal medium for an unknown reason. The absence of toxicity in solid medium is also yet to be understood. Similarly, variations in 4dPN sensitivity between rich versus minimal medium and liquid versus solid medium can be detected in several mutants of *de novo* PLP biosynthesis as well as the *yggS* B_6_ homeostasis mutant. While beyond the scope of this study, we find these variations to be interesting and believe they provide a foundation for future B_6_ homeostasis-related studies.

Using vitamin B_6_ auxotrophs we have shown that, in agreement with previous findings, 4dPN is taken up from the medium and is able to competitively inhibit cumulative PN uptake [19]. Genetic experiments and HPLC analysis led us to confirm that the 4dPN sensitivity phenotype seen in the auxotrophic strains results from the loss of B_6_ kinase activity. Further analysis showed that 4dPN inhibits PdxK specifically, but does not appear to interfere with the activity of PdxY, allowing the auxotrophs to overcome 4dPN sensitivity in the presence of PL.

We have also shown that like PN, 4dPN is rapidly phosphorylated by PdxK to produce 4dPNP. Additionally, like PN and PNP, 4dPN and 4dPNP inhibit activity of important PLP/PNP-binding enzymes. In agreement with previous publications [36], we found that 4dPN inhibits PdxH activity. A recent publication studying 4dPN toxicity in *Salmonella enterica *, a close relative of *E. coli *, reported similar findings [38]. Additionally, the authors propose a model in which GlyA or the GCV system may be inhibited by 4dPNP, reducing CoA synthesis and ultimately thiamine levels. In agreement with this proposed model, we have found that both PNP and 4dPNP inhibit activity of the highly prevalent PLP-dependent enzyme GlyA *in vitro*. This enzyme catalyses the conversion of the essential cofactor tetrahydrofolate (THF) to 5, 10-methyl-tetrahydrofolate (5, 10-mTHF) for one-carbon unit biosynthesis. In a *glyA* mutant, the glycine cleavage system (GCV) can catalyse a similar reaction, fulfilling this metabolic need. GcvP, the PLP-dependent enzyme of GCV, has recently been shown to be inhibited by PNP [18]. While inhibition of GcvP has not been tested to our knowledge, it’s possible that like GlyA, GcvP activity may also be inhibited by 4dPNP and if so, inhibition of both routes of 5, 10-mTHF production in the presence of both PN and 4dPN could indeed partially explain the 4dPN sensitivity phenotype seen in wild-type during growth in rich medium [38].

A large portion of our study focused on the 4dPN sensitivity of the Δ*yggS* mutant. Deletion of *yggS* leads to disrupted vitamin B_6_ homeostasis and the mutant has been shown to accumulate intracellular PNP [17]. This PNP accumulation is exacerbated by supplementation with exogenous PN, resulting in a visible ‘PN toxicity’ phenotype [4]. In this study, we observed a mild 4dPN sensitivity phenotype in the Δ*yggS* mutant that is greatly exacerbated by supplementation with PN. Interestingly, experimental plate phenotypes show that the ratio of 4dPN to PN is crucial to the severity of 4dPN toxicity to Δ*yggS* and this finding is supported by Tn-Seq data. The Δ*yggS* mutant does not exhibit sensitivity to PL or PM alone, but we showed that supplementation with specific concentrations of these vitamers also influences the 4dPN sensitivity of the mutant. Additionally, disruption of PL phosphorylation in a Δ*yggS* background (Δ*yggS*Δ*pdx*Y double mutant) resulted in increased 4dPN sensitivity. Through analysis of the most recent models of vitamin B_6_ salvage (Fig. 8b), we show that the severe 4dPN sensitivity of a Δ*yggS* mutant is at least in part, the result of an accumulation of both PNP and 4dPNP, likely resulting in the inhibition of important PLP-dependent enzymes.

While we hypothesize that the 4dPN sensitivity of the *yggS* mutant is due to the accumulation of and inhibition by both 4dPNP and PNP, the mechanism behind buildup of PNP in the *yggS* mutant is yet to be understood. As this sensitivity is exacerbated in the Δ*yggS* Δ*pdxY* mutant (Fig. 8), it’s possible that like PdxY, YggS plays a role in conversion between PL and PLP.

Elimination of PdxA activity should result in decreased intracellular PNP concentrations through disruption of *de novo* PLP biosynthesis, therefore we hypothesized that 4dPN sensitivity would decrease in a Δ*yggS*Δ*pdxA* mutant compared to the Δ*yggS* mutant. While this hypothesis was confirmed when supplemented with exogenous PL, we found that 4dPN sensitivity was unexpectedly exacerbated in the Δ*yggS*Δ*pdxA* mutant in the presence of PN (Fig. 8c). This result is not intuitive with the current knowledge and models of vitamin B_6_ biosynthesis, salvage, and homeostasis in *E. coli* and may be a clue pointing toward regulatory mechanisms of the complicated maintenance of B_6_ vitamer homeostasis.

In this study we have characterized the sensitivity response of wild-type *E. coli* and several important mutants of PLP biosynthesis, salvage, and homeostasis and have further solidified the importance of *yggS* in vitamin B_6_ homeostasis. As 4dPN perturbs several PLP-related processes it can serve as a useful compound to further characterize the components of vitamin B_6_ metabolism in *E. coli* that are still not understood, such as the molecular function of YggS, the regulation of PLP synthesis and salvage genes, the role of orphan PLP-dependent regulators and the missing B_6_ transporters [5].

## Supplementary Data

Supplementary material 1

Supplementary material 2

Supplementary material 3
