# Supplementary material for: 4′-Deoxypyridoxine disrupts vitamin B6 homeostasis in Escherichia coli K12 through combined inhibition of cumulative B6 uptake and PLP-dependent enzyme activity
Source: Microbiology (Reading). 2023 Apr 11;169(4):001319. doi: 10.1099/mic.0.001319 (PMC10202323; doi:10.1099/mic.0.001319)
Supplement: Supplementary material 1 [file mic-169-1319-s001.pdf]

Supplemental tables, results and figures

Table S1. Strains and plasmids used in this study

Strain, plasmid	Genotype and/or description	Ref/Source
BW25113 (wild-type)	F-; Δ(araD-araB)567; ΔlacZ4787(::rrnB-3); λ-; rph-1; Δ(rhaD-rhaB)568; hsdR514	(Baba et al., 2006)
DH5α	F– ϕ80lacZΔM15 Δ(lacZYA-argF)U169 recA1 endA1 hsdR17(rK–, mK+) phoA supE44 λ–thi-1 gyrA96 relA1	NEB
JW2317	Δ*pdxB::kan*	(Baba et al., 2006)
JW2548	Δ*pdxJ::kan*	(Baba et al., 2006)
JW2411	Δ*pdxK::kan*	(Baba et al., 2006)
JW1628	Δ*pdxY::kan*	(Baba et al., 2006)
JW0051	Δ*pdxA::kan*	(Baba et al., 2006)
JW1403	Δ*ydbC* (Δ*pdxI::kan*)	(Baba et al., 2006)
JW2894	Δ*epd::kan*	(Baba et al., 2006)
JW0890	Δ*serC::kan*	(Baba et al., 2006)
JW0749	Δ*ybhA::kan*	(Baba et al., 2006)
JW1630	Δ*pdxH::kan*	(Baba et al., 2006)
NU812	Δ*pdxA::kan*	(Roa et al., 1989)
VDC6594	BW25113 ΔyggS::frt	(Prunetti et al., 2016)
VDC9816	BW25113 ΔpdxB::frt ΔpdxJ::kan	This study
JTB1038	BW25113 ΔpdxB::frt ΔpdxJ::frt	This study
JTB1134	BW25113/pBAD24	This study
JTB1136	BW25113/pBAD24::yggS	This study
JTB1142	BW25113 ΔyggS::frt/pBAD24	This study
JTB1144	BW25113 ΔyggS::frt/pBAD24::yggS	This study
JTB1324	BW25113 ΔpdxK::kan	This study
JTB1326	BW25113 ΔpdxY::kan	This study
JTB1394	BW25113 ΔpdxA::kan	This study
JTB1397	BW25113 ΔpdxI::kan	This study
JTB1400	BW25113 Δepd::kan	This study
JTB1403	BW25113 ΔserC::kan	This study
JTB1406	BW25113 ΔybhA::kan	This study
JTB1409	BW25113 ΔpdxK::frt	This study
JTB1411	BW25113 ΔpdxY::frt	This study
JTB1413	BW25113 ΔpdxK::frt ΔpdxY::kan	This study
JTB1417	BW25113 ΔyggS::frt ΔpdxY::kan	This study
JTB1431	BW25113 ΔpdxH::kan	This study
JTB1437	BW25113 ΔyggS::frt ΔpdxI::kan	This study

JTB1440	BW25113 ΔpdxY::frt ΔpdxI::kan	This study
JTB1443	BW25113 ΔyggS::frt ΔpdxY::frt	This study
JTB1446	BW25113 ΔyggS::frt ΔpdxY::frt ΔpdxI::kan	This study
JTB1485	BW25113 ΔyggS::frt ΔpdxA::kan	This study
JTB1488	BW25113 ΔyggS::frt ΔpdxK::kan	This study
JTB1491	BW25113 ΔyggS::frt ΔpdxK::kan/pBAD24	This study
JTB1494	BW25113 ΔyggS::frt ΔpdxK::kan/pBAD24::yggS	This study
JTB1497	BW25113 ΔyggS::frt ΔpdxK::frt	This study
JTB1590	BW25113 ΔpdxK::frt ΔpdxY::frt ΔpdxI::kan	This study
JTB1612	BW25113 ΔpdxY::frt ΔpdxA::kan	This study
JTB1615	BW25113 ΔpdxK::frt ΔpdxA::kan	This study
**Plasmids**		
pCP20	Yeast FLP recombinase Cm^R Amp^R oriR101 w/ repA101ts	(Cherepanov and Wackernagel, 1995)
pBAD24	Amp^R, ColE1, arabinose inducible promoter	(Guzman et al., 1995)
pBY291.3	pBAD24::*yggS*$_{EC}$ (Amp^R, ColE1)	(Prunetti et al., 2016)

Table S2. Primers used in this study

Primer	Alias	Sequence (5'->3')	Purpose
DH596	BADyggS_fwd	TATATACCATGGACGATATTGCGCATAACCTGGC	Construction of pBY291.3
DH598	BADyggS_rev	TTAATTAAGCATGCTTATTTTTTAGAGTAATCACGCGC	Construction of pBY291.3
JTBP112	serC_ext_F	GTGCAAATCCAGAATGGACG	Confirmation of P1 transduction success
JTBP113	serC_ext_Rev	GTTTTGCCGTGTGCTAATGC	Confirmation of P1 transduction success
JTBP114	serC_int_F	AGTGACTGTTGATGGTCTGC	Confirmation of P1 transduction success
JTBP115	serC_int_Rev	AGTCTGTCAGCGCTTTAACG	Confirmation of P1 transduction success
JTBP116	pdxA_ext_F	ATACCGCATGCTGATGAACC	Confirmation of P1 transduction success
JTBP117	pdxA_ext_Rev	TGTCGATCACGAACTGATCG	Confirmation of P1 transduction success
JTBP118	pdxA_int_F	TTAACGCTACTTCCTGTCGC	Confirmation of P1 transduction success
JTBP119	pdxA_int_Rev	TTGATGGCGAGATTAAGCGC	Confirmation of P1 transduction success
JTBP120	epd_ext_F	TCGCTTATCCTAGCTGAAGC	Confirmation of P1 transduction success
JTBP121	epd_ext_Rev	AGACATGGTGAATCCTCTCG	Confirmation of P1 transduction success
JTBP122	epd_int_F	ATCCGCGTATTGCATGAACG	Confirmation of P1 transduction success
JTBP123	epd_int_Rev	TGAAATGCACCTTGTGCTGC	Confirmation of P1 transduction success

JTBP124	pdxY_ext_F	AAACTATCGCCTCCAATGCC	Confirmation of P1 transduction success
JTBP125	pdxY_ext_Rev	TATGTGAACGAGGCACTGAAGG	Confirmation of P1 transduction success
JTBP126	pdxY_int_F	ACACCCAATACGGCAAATGG	Confirmation of P1 transduction success
JTBP127	pdxY_int_Rev	TGATATGCCAGGCTTCATCG	Confirmation of P1 transduction success
JTBP128	pdxK_ext_F	AATCGATGACGACGAAGCAACC	Confirmation of P1 transduction success
JTBP129	pdxK_ext_Rev	TGAACTGTTCGTCCACTTCG	Confirmation of P1 transduction success
JTBP130	pdxK_int_F	AACGGCCTGAATGTCTTTGC	Confirmation of P1 transduction success
JTBP131	pdxK_int_Rev	AATGACATTCACGCTGTCGG	Confirmation of P1 transduction success
JTBP132	pdxH_ext_F	AACCGATACGCTGGAATACC	Confirmation of P1 transduction success
JTBP133	pdxH_ext_Rev	ATAACGACTGCACCTTTGCC	Confirmation of P1 transduction success
JTBP134	pdxH_int_F	AATACACCAAAGGCGGGTTACG	Confirmation of P1 transduction success
JTBP135	pdxH_int_Rev	TCAATCTGTTCAAGGCTGACGC	Confirmation of P1 transduction success
JTBP136	yggS_ext_F	AATCCAGCTGACCATTCTCC	Confirmation of P1 transduction success
JTBP137	yggS_ext_Rev	GAATATAGGCAACCAGCAGCG	Confirmation of P1 transduction success
JTBP138	yggS_int_F	ATCGCAGAAGCCATTGATGC	Confirmation of P1 transduction success
JTBP139	yggS_int_Rev	TCCATATCGTCCGACATTCCC	Confirmation of P1 transduction success
JTBP140	pdxI_ext_F	CACTTTAACGTCGATGACGG	Confirmation of P1 transduction success
JTBP141	pdxI_ext_Rev	AAACGTATCCAGCCGCAATTCC	Confirmation of P1 transduction success
JTBP142	pdxI_int_F	AATCAGATTATCCGCGAAGCGC	Confirmation of P1 transduction success
JTBP143	pdxI_int_Rev	AAAGTGTGGACGATTGCAGC	Confirmation of P1 transduction success
JTBP144	ybhA_ext_F	AGTGGTATCGGTGTACCATTCG	Confirmation of P1 transduction success
JTBP145	ybhA_ext_Rev	ATGAACTGGCGATCAAACCTGG	Confirmation of P1 transduction success
JTBP146	ybhA_int_F	TAATCATCGTCACAGGTCGC	Confirmation of P1 transduction success
JTBP147	ybhA_int_Rev	AAAGTTATCGCCGAATGCCACG	Confirmation of P1 transduction success
JTBP148	glyA_ext_F	TTAGCTGAGTCAGGAGATGC	Confirmation of P1 transduction success
JTBP149	glyA_ext_Rev	AAATTGCCTGATGCGCTACG	Confirmation of P1 transduction success
JTBP150	glyA_int_F	AGAGCACATCGAACTGATCG	Confirmation of P1 transduction success
JTBP151	glyA_int_Rev	ATCATTGATGCTGTCCAGCACG	Confirmation of P1 transduction success

Supplemental Results: Growth and 4dPN-sensitivity characterization of mutants of PLP biosynthesis, salvage, and PLP-dependent enzymes

Mutants of PLP biosynthesis, salvage, and PLP-dependent enzymes have general growth characteristics that differ from wild-type

During analysis of 4dPN sensitivity in liquid media, results revealed several interesting growth characteristics seen in PLP-related mutants that differed from wild-type growth, in the absence of 4dPN or unaffected by its presence.

All auxotrophic strains exhibited a significantly longer lag-phase than the wild-type strain but, with the exception of Δ*pdxH* (which is known to have generally defective growth (1)) reached a higher final cell density during growth in rich medium without 4dPN (**Fig. S5A**).

Similarly, Δ*epd*, Δ*pdxI*, and Δ*ybhA* appear to have a significantly increased lag phase, but ultimately reach a higher final cell density than wild-type when untreated or treated with low doses of 4dPN in rich medium (**Fig. S5B**). As seen in rich medium, Δ*pdxI* and Δ*ybhA* reach a higher final cell density when untreated or treated with 4 mM or less 4dPN, but both mutants have a decreased lag time versus wild-type in minimal medium both with and without 4dPN treatment (**Fig. S5C**). As in rich medium, Δ*epd* reaches a higher final cell density than wild-type but has a similar growth rate in untreated minimal medium.

Alternatively, both kinase mutants (Δ*pdxK* and Δ*pdxY*) appear to have similar growth to wild-type *E. coli* in untreated rich medium (**Fig. S5B**), but in liquid minimal medium Δ*pdxK* exhibits a general growth defect versus wild-type, with an increased lag phase and lower final cell density (**Fig. S5C**). Δ*pdxY* has a similar growth rate to wild-type in minimal medium but exhibits a growth advantage not seen in the mutant in rich medium, as it outgrows

wild-type, reaching a higher final cell density and is insensitive to 4dPN concentrations below 8 mM (**Fig. S5C)**.

Mutants of PLP biosynthesis, salvage, and PLP-dependent enzymes have varied sensitivity to 4dPN

Results reported in this study show a connection between 4dPN toxicity and inhibition of cumulative PN uptake, resulting in growth inhibition of B_6 auxotrophic strains. As 4dPN sensitivity also occurs wild-type and other mutants that are capable of *de novo* PLP biosynthesis, low intracellular B_6 concentrations are unlikely to be the cause of 4dPN sensitivity of non-auxotrophic strains. Tn-Seq analysis also identified fitness changes of both auxotrophic and non-auxotrophic PLP biosynthesis and salvage mutants grown in the presence of PN and 4dPN, but few of these fitness changes were significant (**Fig. S6A**). However, a strong fitness defect was seen in the *serC* mutant in minimal medium that was exacerbated by the presence of 4dPN/PN, as well as in mutants of important PLP-dependent enzymes, such as TrpB and HisC (**Fig. S6B)**.

The 4dPN sensitivity of the *yggS* mutant is of particular interest, as it doesn't exhibit a standard dose-dependent sensitivity phenotype. which is capable

In vivo 4dPN sensitivity assays of a non-auxotrophic mutant of PLP biosynthesis (Δ*epd*) and of different salvage mutants (Δ*pdxI*, Δ*pdxK*, Δ*pdxY*, and Δ*ybhA*) revealed that several mutants are more sensitive to 4dPN than wild-type *E. coli*, but the phenotypes differ between mutants.

As seen with the B_6 auxotrophic strains, the presence of 4dPN leads to a concentration dependent zone of growth inhibition of the Δ*epd* mutant on rich medium plates (**Fig. S7A**). Unlike the auxotrophic mutants, Δ*epd* was not sensitive to 4dPN on minimal medium plates in the presence or absence of B_6 vitamers (data not shown).

Of the salvage mutants, only Δ*pdxY* was sensitive to 4dPN on solid rich medium, with growth of the other mutants (Δ*pdxI*, Δ*pdxK*, and Δ*ybhA*) resembling wild-type. Interestingly, the phenotype exhibited by the Δ*pdxY* mutant resembles that of Δ*yggS*, with a concentration-dependent ring of growth inhibition (**Fig. S7A**). Like the wild-type and Δ*epd* strains, the salvage mutants do not exhibit sensitivity to 4dPN on minimal medium plates in the presence or absence of B_6 vitamers (data not shown).

4dPN sensitivity assays in liquid medium gave somewhat uncorrelated results. Assays in rich medium revealed that like wild-type, all non-auxotrophic strains follow a standard concentration-dependent sensitivity to 4dPN with increasing concentrations resulting in increasing growth inhibition (**Fig. S5B**). In liquid minimal medium both kinase mutants are fairly insensitive to 4dPN, with no growth defects observed in Δ*pdxK* with less than 4 mM 4dPN and Δ*pdxY* with 4dPN concentrations below 8 mM (**Fig. S5C)**.

As seen in rich medium, Δ*pdxI* and Δ*ybhA* exhibit a standard dose-dependent sensitivity to 4dPN in liquid minimal medium (**Fig. S5C**). Interestingly, the 4dPN sensitivity of Δ*epd* in liquid minimal medium resembles that of the *yggS* mutant, with both the lowest and highest concentrations of 4dPN leading to the greatest growth inhibition (**Fig. S5C)**.

Supplementary Discussion

In liquid media, Δ*yggS* and Δ*epd* have similar 4dPN sensitivity phenotypes, with a clear non-traditional, concentration-dependent pattern of sensitivity in minimal medium (**Fig. S5C**). However, the sensitivity phenotypes of Δ*yggS* and Δ*epd* differ on solid media. Unlike Δ*yggS*, Δ*epd* is not sensitive to 4dPN on minimal medium plates in the presence nor absence of B_6 vitamers. On rich medium plates both mutants are sensitive to 4dPN, but the resultant sensitivity phenotype of Δ*epd* resembles that of B_6 auxotrophs with a central zone of growth inhibition (**Fig. S7**), rather

than a concentration dependent ring of growth inhibition as seen in the *yggS* mutant. Δ*epd* is the only mutant of *de novo* PLP biosynthesis that is not auxotrophic for B_6 vitamers. The ability of *E. coli* to overcome the loss of Epd activity is due to the low affinity D-erythrose 4-phosphate dehydrogenase activity of GapA (2). GapA is a high affinity D-glyceraldehyde 3-phosphate dehydrogenase and typically plays a role in glycolysis and gluconeogenesis. The ability of 4dPN to induce an auxotroph-like sensitivity phenotype in the Δ*epd* mutant on rich medium plates but not minimal medium suggests that the availability of central metabolites may have an influence on *de novo* PLP biosynthesis.

Supplemental References

1. Lam HM, Winkler ME. Characterization of the complex pdxH-tyrS operon of Escherichia coli K-12 and pleiotropic phenotypes caused by pdxH insertion mutations. J Bacteriol. 1992/10/01. 1992;174(19):6033–45.
2. Yang Y, Zhao G, Man T-K, Winkler ME. Involvement of the gapA-and epd (gapB)-Encoded Dehydrogenases in Pyridoxal 5-Phosphate Coenzyme Biosynthesis in Escherichia coli K-12 [Internet]. Vol. 180, JOURNAL OF BACTERIOLOGY. 1998 [cited 2019 May 2]. Available from: https://www.ncbi.nlm.nih.gov/pmc/articles/PMC107430/pdf/jb004294.pdf

Supplemental Figures

Figure S1

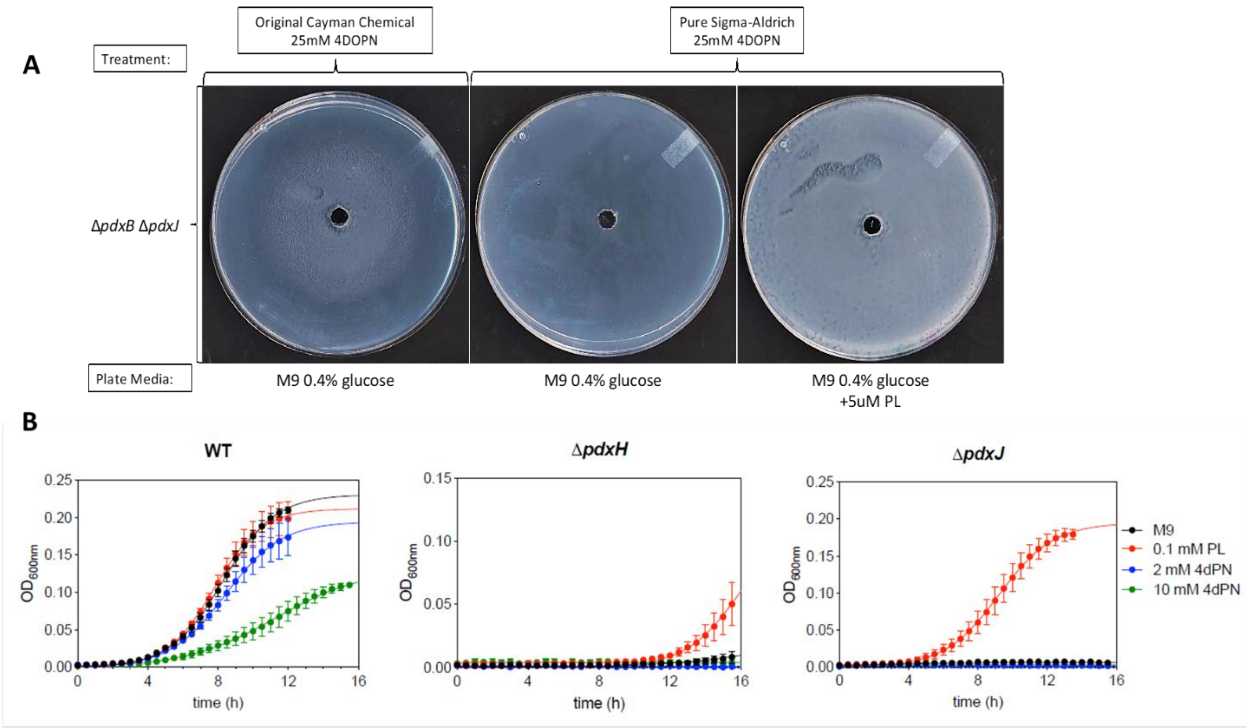

Figure S1. 4-Deoxypyridoxine cannot be utilized by *Escherichia coli* as a source of vitamin B_6. 4dPN sensitivity assays were performed as described in the text. **(A)** growth of a vitamin B_6 auxotroph, Δ*pdxB* Δ*pdxJ*, was analyzed on minimal media plates with or without 5 µM PL when treated with stock solutions of 4dPN purchased from either Cayman Chemical or Sigma-Aldrich. The 4dPN purchased from Sigma-Aldrich was confirmed to be pure by HPLC (Safo lab, VCU). When plated on minimal media treated with 20µL 25 mM 4dPN in central well, the auxotroph was able to utilize the PN analog from Cayman Chemical as a source of B_6 but not from the pure Sigma-Aldrich stock, suggesting that the Cayman Chemical 4dPN stock solution was contaminated with alternate B_6 vitamers. When plated on M9 minimal media supplemented with PL the auxotroph was able to grow (right plate) when treated with 4dPN from Sigma-Aldrich, confirming that the lack of growth with pure 4dPN treatment (center plate) is the result of unavailability of B_6. In all subsequent experiments pure 4dPN purchased from Sigma-Aldrich was used and auxotrophic strains were consistently unable to utilize it as a source of B_6. Panel **B)** Growth curves of *E. coli* BW25113 (WT), and Δ*pdxJ* and Δ*pdxH* auxotrophic strains were obtained during grown in minimal medium supplemented with PL or 4dPN at the indicated concentrations. Neither

Δ*pdxJ* nor Δ*pdxH* was able to utilize any concentration of 4dPN as a source of B_6 during growth in liquid medium, while wild-type growth was inhibited in a concentration dependent manner by increasing concentrations of 4dPN. Each curve represents the average of 3 independent experiments, each performed in triplicate.

Figure S2

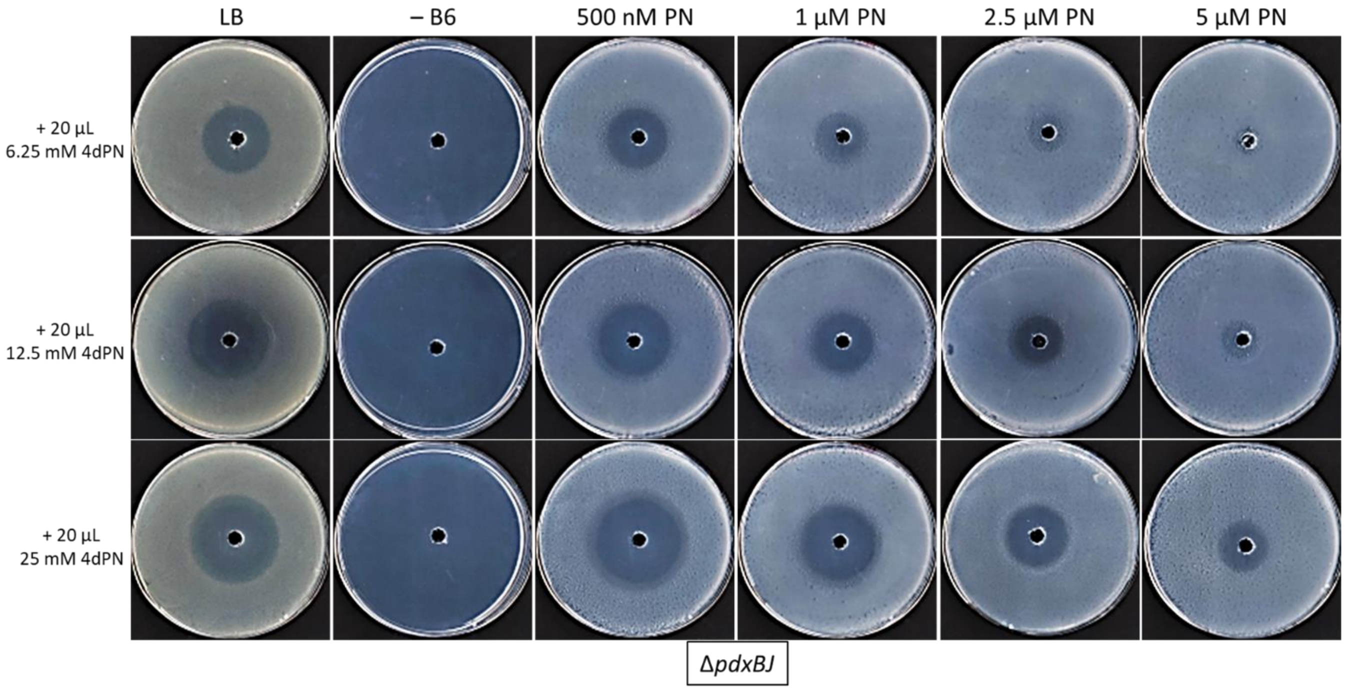

Figure S2. 4-deoxypyridoxine induced growth inhibition of the auxotrophic strains is the result of competitive inhibition of pyridoxine uptake. 4dPN sensitivity assays were performed as described in the text. Exploration of the 4dPN-PN uptake competition was performed by testing a range of 4dPN treatment concentrations on minimal medium plates supplemented with a range of PN concentrations. This experiment confirmed that at the highest ratio of 4dPN treatment to PN concentration tested (25 mM 4dPN in a central well of plates supplemented with 500 nM PN) resulted in the largest zone of growth inhibition while the smallest 4dPN treatment to PN concentration tested (6.25 mM 4dPN in a central well of plates supplemented with 5 µM PN)

resulted in essentially no visible 4dPN sensitivity. This finding strongly suggests that the 4dPN sensitivity phenotype observed in vitamin B_6 auxotrophs is the result of competitive inhibition of PN uptake by 4dPN.

Figure S3

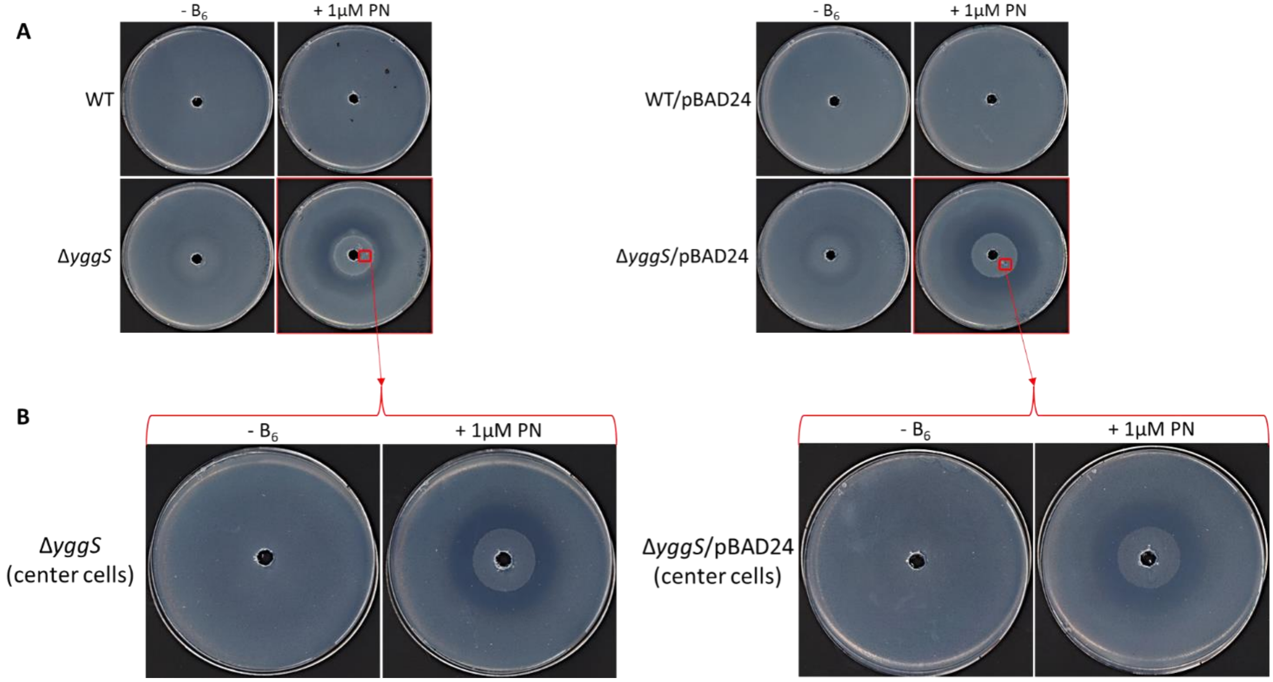

Figure S3. The 4dPN sensitivity ring phenotype of Δ*yggS* is not due to development of suppressor mutations, but rather concentration-dependent toxicity. 4dPN sensitivity assays were performed as described in the text. **(A)** In order to validate the unusual 4dPN sensitivity ring phenotype of Δ*yggS*, cells of the indicated strains were plated on minimal medium plates with and without 1 µM supplemented PN and were treated with 20 µL of 25 mM 4dPN in central wells. Cells were collected from growth in the center of experimental plates exhibiting strong phenotypes (Δ*yggS* and ΔyggS/pBAD24 plated on M9 glucose + 1 µM PN; indicated by red boxes) and used to repeat the plated 4dPN sensitivity assay. **(B)** The experiment using center cells revealed a concentration-dependent ring of 4dPN sensitivity, repeating the original experimental results and confirming that this phenotype is not due to the formation of suppressor mutants in Δ*yggS* at very

high (25 mM) concentrations of 4dPN. Both Δ*yggS* and ΔyggS/pBAD24 strains were tested to rule out the possibilities of antibiotic or vector influence on the 4dPN sensitivity phenotype.

Figure S4

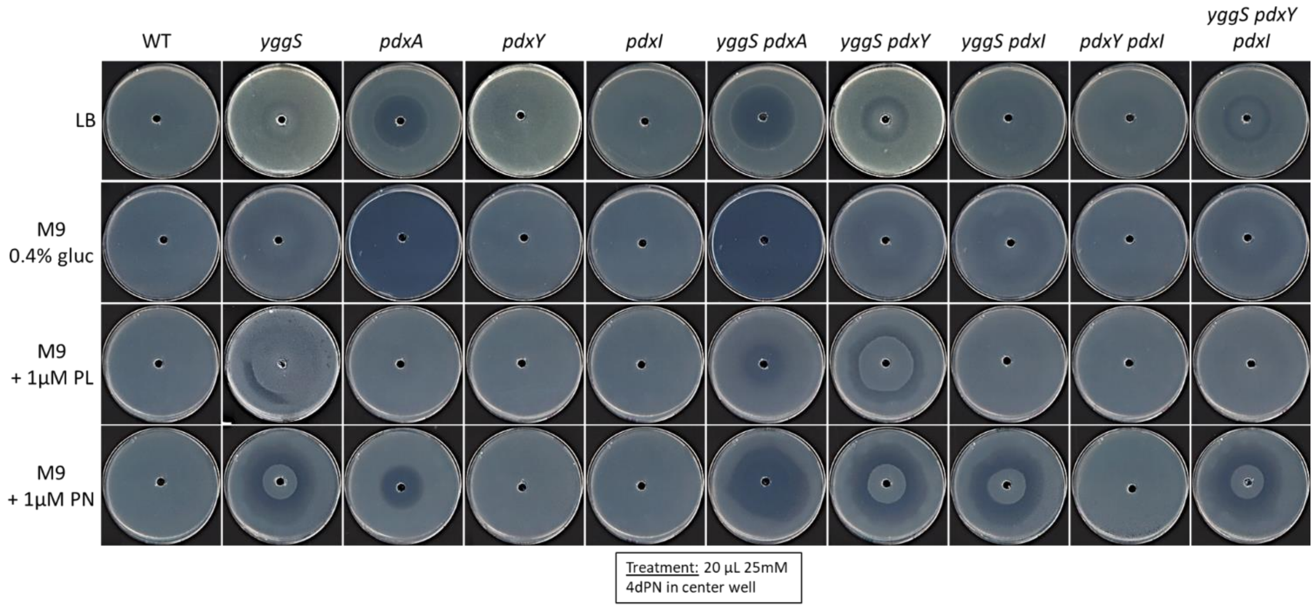

Figure S4. The 4dPN sensitivity phenotype of Δ*yggS* is the result of accumulated PNP and 4dPNP. 4dPN sensitivity assays were performed as described in the text. 4dPN sensitivity assays were carried out for single and multiple mutants of PLP-related genes involved in production and accumulation of PNP on rich medium and minimal medium with and without supplemented 1µM PL or PN. Plated bacterial cells (OD_{600} = 0.006) were treated with 20 µL of 25 mM 4dPN in a central well. All plate sensitivity experiments were repeated at least three times.

Figure S5

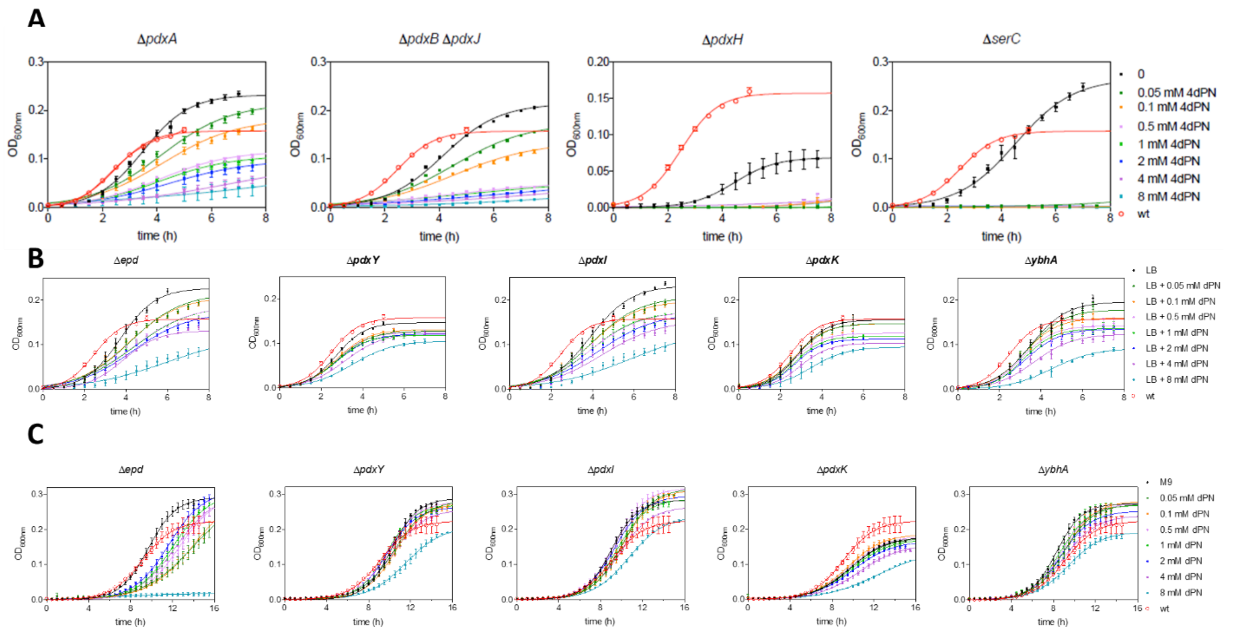

Figure S5 . *E. coli* mutants of PLP homeostasis, biosynthesis and salvage have growth characteristics that differ from wild-type (A) Growth curves of *E. coli* auxotrophic mutants of the *de novo* PLP biosynthesis pathway (Δ*pdxA*, Δ*pdxB* Δ*pdxJ*, Δ*pdxH*, and Δ*serC*) obtained during grown in rich medium with and without 4dPN treatment. All auxotrophic strains have a prolonged lag phase, but ultimately reach a higher final OD600 than wild-type (wt) when untreated or treated with low concentrations of 4dPN (except Δ*pdxH*, which has a known growth defect). **(B)** Growth curves of *E. coli* non-auxotrophic mutants of the *de novo* PLP biosynthesis and salvage pathways (Δ*epd*, Δ*pdxY*, Δ*pdxI*, Δ*pdxK*, and Δ*ybhA*) obtained during grown in rich medium with and without 4dPN treatment. **(C)** Growth curves of *E. coli* non-auxotrophic mutants of the *de novo* PLP biosynthesis and salvage pathways (Δ*epd*, Δ*pdxY*, Δ*pdxI*, Δ*pdxK*, and Δ*ybhA*) obtained during grown in minimal medium with and without 4dPN treatment. Each curve represents the average of 3 independent experiments, each performed in triplicate.

Figure S6

A

Table S4. Tn-Seq: Averaged Fitness of PLP-related gene mutants during 4dPN/PN growth challenges							
**PLP-related mutant**		**M9**	**M9 supplemented with excess 4dPN to PN**				**LB**
**Gene**	**Description (NCBI)**	No 4dPN/PN	2.5x	5x	12.5x	250x	No 4dPN/PN
pdxI	predicted oxidoreductase, NAD(P)-binding	-0.03	0.09	0.13	0.15	0.22	0.27
epd	D-erythrose 4-phosphate dehydrogenase	0.24	-0.10	-0.23	-0.34	-0.11	-0.001
ybhA	predicted hydrolase	0.06	0.01	0.05	-0.09	0.14	-0.07
pdxY	pyridoxine kinase	0.24	0.17	-0.21	-0.08	-0.16	0.11
pdxK	pyridoxine kinase	-0.07	-0.83	0.23	-0.40	0.10	-0.07
yggS	predicted PLP homeostasis enzyme	0.61	-5.57	-4.26	-4.60	-4.94	-0.27
serC	phosphoserine aminotransferase	-4.56	-6.30	-4.41	-4.46	-5.20	-0.14
hisC	histidinol-phosphate aminotransferase	-3.72	-5.61	-4.29	-3.52	-5.04	0.16
trpB	tryptophan synthase subunit beta	-4.21	-5.73	-4.96	-5.58	-5.31	-0.09

B

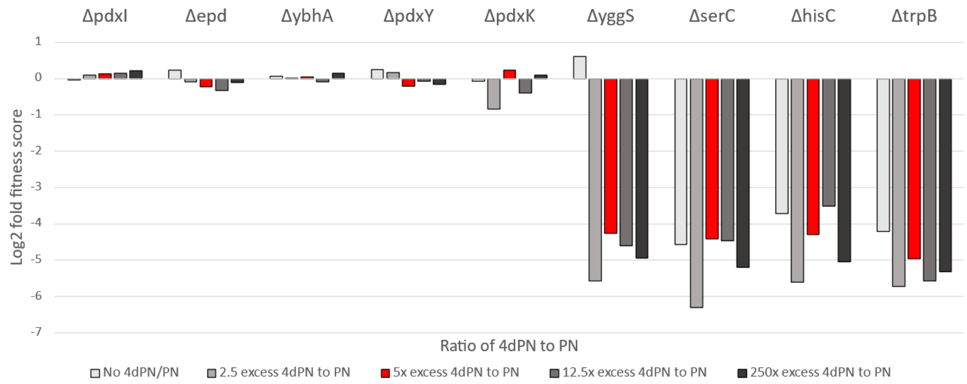

Figure S6. Tn-Seq data from 4dPN/PN challenge assays revealed mutant-specific trends of 4dPN/PN sensitivity that differ by ratio of anti-vitamer to vitamer. (A) Average log2-fold fitness scores (of four replicates) for mutants of PLP biosynthesis, salvage, and of highly impacted PLP-dependent enzymes. **(B)** Comparison of fitness trends in PLP-related mutants challenged with different ratios of 4dPN to PN are shown by averaged (of four replicates) log2-fold fitness scores. While fitness changes of most shown synthesis and salvage mutants are relatively insignificant, large fitness defects were detected in Δ*yggS*, Δ*serC*, Δ*hisC*, and Δ*trpB*. Interestingly, these fitness defects appear to be greatest with the lowest and highest ratios of 4dPN-to-PN, with the smallest fitness defect seen at intermediate ratios (5x 4dPN to PN is labelled in red).

Figure S7

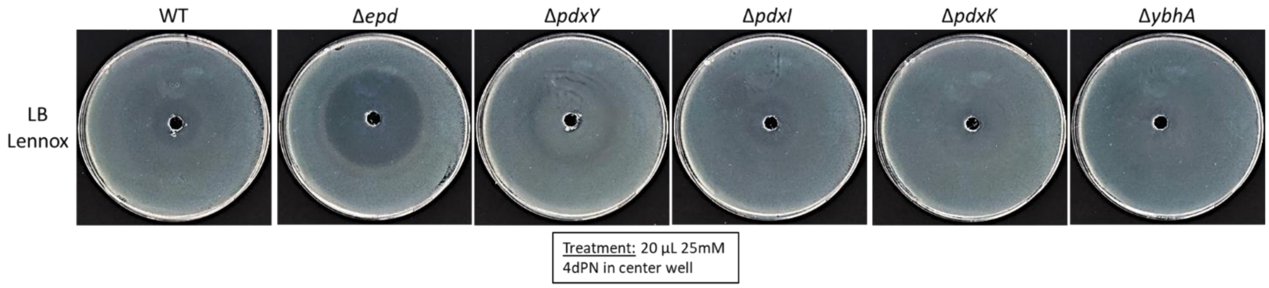

Figure S7. Mutants of PLP homeostasis, biosynthesis and salvage have varying 4-deoxypyridoxine sensitivity phenotype. Plated and liquid 4dPN sensitivity assays of non-auxotrophic mutants of *de novo* PLP biosynthesis and salvage were carried out in rich, low salt (LB-Lennox) medium. Plated bacterial cells (OD_{600} = 0.006) were treated with 20 µL of 25 mM 4dPN in a central well, revealing distinct differences in 4dPN sensitivity phenotypes between mutants.
